# Electron Tomography of Cryofixed, Isometrically Contracting Insect Flight Muscle Reveals Novel Actin-Myosin Interactions

**DOI:** 10.1371/journal.pone.0012643

**Published:** 2010-09-09

**Authors:** Shenping Wu, Jun Liu, Mary C. Reedy, Richard T. Tregear, Hanspeter Winkler, Clara Franzini-Armstrong, Hiroyuki Sasaki, Carmen Lucaveche, Yale E. Goldman, Michael K. Reedy, Kenneth A. Taylor

**Affiliations:** 1 Institute of Molecular Biophysics, Florida State University, Tallahassee, Florida, United States of America; 2 Medical Research Council Laboratory of Molecular Biology, Cambridge, England; 3 Department of Cell Biology, Duke University Medical Center, Durham, North Carolina, United States of America; 4 Pennsylvania Muscle Institute, University of Pennsylvania, Philadelphia, Pennsylvania, United States of America; 5 Division of Fine Morphology, Core Research Facilities, Jikei University School of Medicine, Tokyo, Japan; Sun Yat-Sen University, China

## Abstract

**Background:**

Isometric muscle contraction, where force is generated without muscle shortening, is a molecular traffic jam in which the number of actin-attached motors is maximized and all states of motor action are trapped with consequently high heterogeneity. This heterogeneity is a major limitation to deciphering myosin conformational changes in situ.

**Methodology:**

We used multivariate data analysis to group repeat segments in electron tomograms of isometrically contracting insect flight muscle, mechanically monitored, rapidly frozen, freeze substituted, and thin sectioned. Improved resolution reveals the helical arrangement of F-actin subunits in the thin filament enabling an atomic model to be built into the thin filament density independent of the myosin. Actin-myosin attachments can now be assigned as weak or strong by their motor domain orientation relative to actin. Myosin attachments were quantified everywhere along the thin filament including troponin. Strong binding myosin attachments are found on only four F-actin subunits, the “target zone”, situated exactly midway between successive troponin complexes. They show an axial lever arm range of 77°/12.9 nm. The lever arm azimuthal range of strong binding attachments has a highly skewed, 127° range compared with X-ray crystallographic structures. Two types of weak actin attachments are described. One type, found exclusively in the target zone, appears to represent pre-working-stroke intermediates. The other, which contacts tropomyosin rather than actin, is positioned M-ward of the target zone, i.e. the position toward which thin filaments slide during shortening.

**Conclusion:**

We present a model for the weak to strong transition in the myosin ATPase cycle that incorporates azimuthal movements of the motor domain on actin. Stress/strain in the S2 domain may explain azimuthal lever arm changes in the strong binding attachments. The results support previous conclusions that the weak attachments preceding force generation are very different from strong binding attachments.

## Introduction

The conversion of the chemical energy of ATP into mechanical work by myosin involves coordinated changes in the actomyosin affinity and the orientation of myosin cross-bridges relative to the fiber axis [1]. Generally, one or more weak binding intermediates, referred to as A-states, precede strong binding states, referred to as R-states, which produce filament sliding [2]. A leading model describing these conformational changes evolved initially from spectroscopic evidence combined with structural information available at the time [3] with later support from the atomic structures of myosin subfragment 1 (S1) [4], [5] and of the actin filament [6]. This model incorporates the concept that the actin-binding motor domain (MD) of myosin maintains a single, stereospecific orientation when actin-bound in the strong binding configuration. The second major domain of myosin is the lever arm, which consists of the myosin converter domain, essential and regulatory light chains, and their bound α-helical heavy chain segment. The working stroke is produced by lever arm rotation about a pivot point near the ATP binding site [7], [8].

Differences in A-states and R-states predominately involve the configuration of a long cleft that divides the myosin MD into upper and lower 50 kDa subdomains, the so-called actin binding cleft. A-states, which have weak actin affinity, have an open cleft while R-states, which bind strongly to actin, have a closed cleft [1]. The lever arm orientation of A-states can be either “up” in a pre-working-stroke position or “down” in a post-working-stroke position; R-states are lever-arm-down states. This model is supported by crystal structures of various isoforms and types of myosin S1 with different nucleotides bound [9]–[13] and when strongly bound to actin in vitro [5], [7], [14], [15].

The structural changes that occur in the A- to R-state transition are poorly defined, especially for myosin heads operating in situ. In the model described above, A-state myosin heads search for the myosin binding site on actin through a rapid equilibrium between attached and detached states until the MD alights on the myosin binding site on actin in the correct orientation for cleft closure. An alternative model involves diffusion of the myosin head on actin to the correct location and orientation for strong binding [16], [17]. The two models produce differences in the potential size of the working stroke.

Working strokes of 10–12 nm are about the maximum that can be achieved by a purely axial motion of the myosin lever arm and have been observed for single myosin S1 molecules *in vitro* [18]. However, working strokes much larger have also been reported [16]. To achieve longer working strokes requires either axial rotation of the MD during the working stroke [19], which can produce a small increase in working stroke, or diffusion of the MD along the actin filament by one or more subunits which can produce much larger working strokes [20]. Several observations suggest that the initial weak binding actin-myosin interaction changes in structure or orientation on actin during tension development. X-ray diffraction of frog muscle [21], [22] indicates that tension development involves a stabilization of the MD from a disordered actin attachment to an ordered one. A disordered to ordered transition of attached cross-bridges is suggested by electron paramagnetic resonance of spin labelled MDs [23]. However, diffusion of the myosin head along actin as a means to increase the working stroke remains controversial, especially if it is considered that filament movement might require a strongly bound myosin attachment.

Visualizing active cross-bridges, including those bound to actin in the weak binding states thought to precede strongly bound force producing states, is essential for defining the structural transitions that constitute the working stroke of myosin. Because of their low actin affinity and possible heterogeneous structure when attached to actin, weakly-bound states are difficult to trap in vitro in numbers with sufficient homogeneity to be amenable to direct visualization by any of the powerful averaging techniques of cryoEM. However, 3-D visualization can be achieved using the technique of electron tomography (ET) which is capable of imaging individual molecules within a highly heterogeneous ensemble [24]. ET has produced 3-D images of insect flight muscle (IFM), including the variable conformations of in situ cross-bridges in rigor [25], [26], in a weakly-bound equilibrium state produced by adenylyl-imidodiphosphate (AMPPNP) and ethylene glycol [27], [28] as well as in snapshots of actively contracting IFM fibers [19].

IFM displays two levels of contraction depending on [Ca^2+^]. Stretch activation, which is characterized by rapid alternating contractions of antagonist muscles during flight, is the contraction mode most often studied [29]. Stretch activation can be induced in skinned fibers at pCa <6.0 [30]. IFM also produces sustained isometric contractions at pCa <4.5, which we refer to as isometric high static tension or iso-HST, that correspond to an isometric tetanus in vertebrate muscle. *In vivo*, iso-HST occurs during the thermogenic “shivering” of preflight warmup [31], when opposing flight muscles contract simultaneously and isometrically to raise the muscle temperature to 40°C where flight can be sustained.

Active myosin heads interact with actin independently of each other so a snapshot of contracting muscle reveals the structure of multiple acto-myosin states within the context of the muscle lattice. Snapshots previously obtained from isometrically activated vertebrate striated muscle revealed a wide range of attachment angles in projections [32]–[34], but these cross-bridges were not visualized in 3-D where detailed interpretation in terms of atomic structure would be possible.

Like the results obtained from vertebrate muscle, iso-HST cross-bridges visualized for the first time by ET also showed a wide range of attachment angles which could be ordered into a sequence compatible with a progressive 13 nm working stroke [19]. Averages computed along axial columns equivalent to the 116 nm long lattice repeat common to both the actin and myosin filaments revealed that actin binding of active cross-bridges was restricted to limited thin filament segments termed “actin target zones” as previously recognized in rigor [35]. Target zones of IFM are positioned midway between successive regulatory complexes which are composed of the three troponin (Tn) peptides. Subsequently, the distribution and orientation of attached cross-bridges from these same tomograms suggested that, in the absence of filament sliding, the variably angled cross-bridge attachments become locally stabilized in each target zone [36]. This observation in turn suggested that individual tension-generating cross-bridges can cycle with little axial translocation or change in axial lever arm angle.

Here we report a more detailed view of the rich variety of myosin head forms in the iso-HST state resulting from improvements in both data collection and analysis that have increased the resolution by 2.5× over the earlier work. The helical arrangement of actin subunits is now resolved, facilitating assignment of particular cross-bridge forms to specific actin subunits within the 38.7 nm repeat that spans from one Tn complex to the next. Multivariate data analysis (MDA) and classification of 3-D repeats is used to quantify individual cross-bridge forms from the number of repeats within each class [37]. Identification of strong and weak binding cross-bridge forms is greatly improved revealing some novel thin filament attachments not previously detected. This leads to a more sharply defined set of cross-bridge structures and interactions in a tension generating muscle than has been possible previously.

## Results

Advancements in data collection and analysis for ET since the iso-HST state was first reported [19] suggested that now is an opportune moment to reexamine this state as a reference for HST specimens subjected to a quick stretch or quick release that are currently under study. The previous data was collected on film with manual adjustment of the tilt angle while the specimen was continuously irradiated and had thus suffered significant radiation damage that at the time was unavoidable. Now, automated tilt series data collection that minimizes radiation damage by using charge coupled device cameras, highly accurate motorized goniometers and computer tracking has made routine the collection of tilt series from even frozen hydrated biological material [38]. Although the specimen blocks used for the present study were the same as those used previously, the improvement in detail is striking.

### Structure of Reassembled 38.7 nm Repeats

Structural analysis of active muscle is a challenge because of the presence of different structural and kinetic states of the myosin. The major analytical problem is the identification and grouping of self-similar structures so that averages with improved signal-to-noise ratio can be computed for comparison to much higher resolution structures obtained by crystallography and cryoelectron microscopy. The tomogram encompassed a myac layer, which is a 25–30 nm thick longitudinal section containing alternating myosin and actin filaments, ∼900 nm square containing 23 thin filaments and from which 515 repeat subvolumes containing a complete 38.7 nm axial repeat (hereafter referred to simply as repeats) were obtained. Our procedures used the thin filament centered on the target zone as a common frame of reference for alignment. To solve the problem of identifying groups of self-similar cross-bridge forms within the heterogeneous ensemble, we used MDA and classification of 3-D repeats and applied this procedure 12 separate times each focusing on separate critical regions of the structure. Averaged images obtained from the classification steps are referred to as class averages; those repeats that form the class are referred to as class members. Two applications of MDA focused on the left and right sides of the thin filament; averages derived from this we refer to as primary class averages. All of the structures in and around the target zone described in detail here were obtained from these two applications. Four more MDA applications identified cross-bridges in the region of Tn (dubbed “Tn-bridges”), four others were used to enumerate myosin head attachments on the eight actin subunits bracketing the target zone. Two more applications were used to verify the lever arm placements of primary class averages as well as to estimate the uncertainty in this critical parameter of cross-bridge structure. The approach is described in detail in a separate publication [37] and briefly here in the Methods.

**Figure 1:**
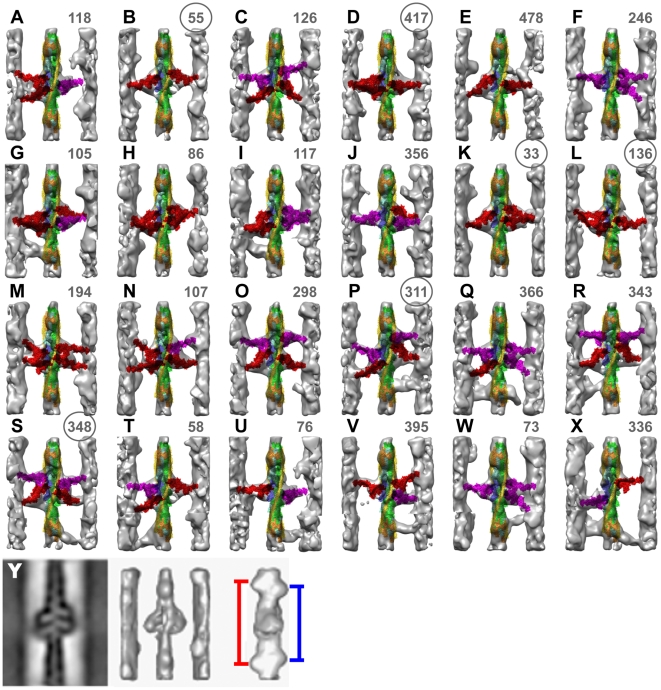
Gallery of reassembled averaged repeats. Each frame is reassembled from left side, right side and Tn-bridge class averages and corresponds to one individual raw repeat whose number is in the upper right hand corner. Circled numbers indicate repeats that are shown at higher resolution in Figure 3. Strong binding cross-bridges (red) and weak binding cross-bridges (magenta) were assigned from the quasiatomic model building. Each variant of primary and Tn-bridge class averages is represented at least once in this gallery. Some are by necessity represented more than once. (A–F) Predominately single-headed bridges, (G–L) mainly 2-headed cross-bridges, (M–R) contains mask motifs and (S–X) Tn-bridges. (Y) This panel shows the central section (left) of the global average after subvolume alignment, surface view (middle) and rotated surface view to reveal the spacing of Tn densities shown by the blue and red lines. Panel Y taken from reference [37].

Column averages in the previous work had an axial resolution of 12.9 nm which is insufficient to reveal the helix of F-actin subunits [19]; here the global average (Fig. 1Y) and all of the reassembled repeats show a zig-zag pattern of density characteristic of F-actin subunits indicating a resolution >5 nm [37]. Significantly, we can now fit into the reconstruction an atomic model of the thin filament independent of any cross-bridge binding. Symmetrically placed about the target zone at the ends of the repeats are densities corresponding to Tn which show a 41.25 nm spacing on one side and 35.75 nm spacing on the other as predicted by the actin helical symmetry (Fig. 1Y).

The multiple different classification steps necessitated a reassembly procedure in which different class averages were combined to make a high signal-to-noise version of each raw repeat. Many different kinds and groupings of cross-bridges were found in the reassembled repeats (Fig. 1). These include 1-headed cross-bridges with lever arms in many different orientations (Fig. 1A–F, J–X), 2-headed cross-bridges, identifiable by the broad mass on the thin filament attributable to the two motor domains (Fig. 1D, G–K), and single heads converging on the target zone from two successive “crowns” on the thick filament (Fig. 1A, C, F, M–T, V, W). This structure is dubbed the mask motif and was first recognized in IFM treated with AMPPNP [27] and later in iso-HST [19].

### Distribution of Myosin Heads along the Thin Filament

We used MDA to separate the different structures, used the number of class members (raw 3-D repeats) to quantify the numbers of cross-bridges of a particular type and used quasiatomic model building to determine whether the interaction was strong or weak (this process is defined below). This data gives a frequency of forming a particular kind of cross-bridge on a specific F-actin subunit within the averaged repeat. The approach is not error free so to obtain some indication of its accuracy, we compared manual counts of cross-bridges with enumerations based on class membership [37]. The RMS deviation of manual enumeration relative to that predicted using MDA was 16% for cross-bridges on the end of the target zone closest to the Z-disk (Z-ward bridges), which are the more frequent types, and 22% for the less frequent bridges on the M-line end (M-ward bridges). Generally, enumeration by class membership overestimates the number of cross-bridges of a particular type because of false positives. Conversely, it may also completely omit some cross-bridges (false negatives) if their structures are too heterogeneous to form a pattern.

**Figure 2:**
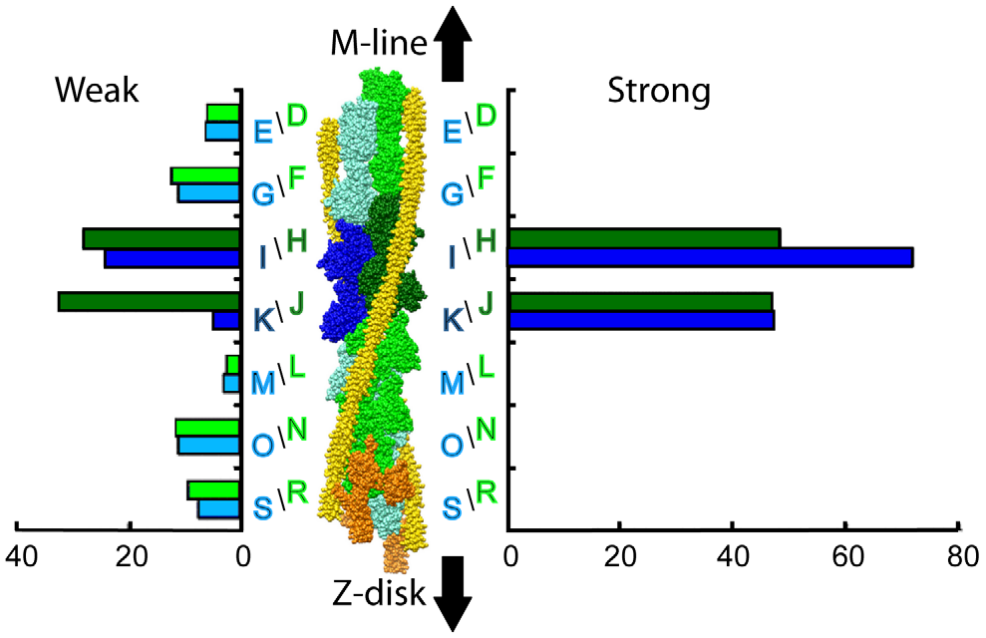
Distributions of myosin heads bound to specific actin subunits on the thin filament. Weak attachments are shown on the left, and strong attachments on the right. The two actin long pitch strands are colored green and blue with the two target-zone actin subunits colored darker shades of green and blue. Occupancy on target-zone actins H–K was obtained from membership of primary class averages. Occupancy on actins R, S was determined from membership of Tn-bridge classification, while occupancy of actin D–G and L–O was determined from special classifications designed for these particular actins. Occupancy of target-zone actins H–K is plotted in darker shades of green and blue. Actin subunit designations correspond to the chain names in the coordinate files deposited in the Protein Data Bank, PDB – 2w49.

The distribution of actin-bound myosin heads is bimodal (Fig. 2). The majority of heads, 78%, are bound to just four F-actin subunits, H–K, two on each side of the actin filament. These are the target-zone actins. Target-zone actins have myosin heads attached 74±16% of the time. Of these, 71% are strong binding attachments that could be generating force and 29% are weak attachments of two general types (the distinction between weak and strong binding is defined below).

The remaining 22% of actin-bound myosin heads are spread over the ten non-target-zone actins for an average frequency of 8±4%, which is to say that 8% of the time these actin subunits have a myosin head bound in some manner. The distribution is not entirely flat but is slightly higher on the two actins on the M-ward side of the target zone (F & G) and on the four actins in the neighborhood of troponin (N, O, R, & S). The frequency of myosin heads bound on the two actins on the Z-ward side of the target zone (L & M) is only 2.6%. This strikingly low number differs by more than 2σ from the average of the other eight non-target-zone actins. The low number of heads on actins L and M is especially significant because it appears right next to the target zone, where both strong and weak myosin binding is highest. The average occupancy of the four troponin actins (N–Q) is 10.3±2.1% which is not significantly different from the average for all non-target-zone actins. Mostly, because of the low frequency of attachments to non-target-zone actins, D, E, L and M, the number of heads on the four actin subunits near troponin appears as a small peak in the distribution. Actins N and O are the location of rear bridges common in rigor muscle, so these actins can accept strong binding myosin interactions, but in iso-HST, surprisingly none were found.

From the number of total repeats, 515, we calculate a corresponding number of thick filament crowns, 458, and a total number of myosin heads potentially available, 3664. Our myosin head counts for all actin attached heads total 1948 heads for 53% attached to actin. The number of strongly bound heads is 1082 representing 29% of the total available. The proportion of strong binding cross-bridges as a fraction of the total available is consistent with measurements from vertebrate striated muscle during isometric contraction [23], [39]–[41].

### Quasiatomic Models of Active Cross-bridges

We built into the global average of all repeats a single atomic model of the thin filament with 28/13 helical symmetry, containing 16 actin subunits, enough tropomyosin (TM) to cover the 16 actins, and four Tn complexes and then used that model for all the reassembled repeats (see Methods). We estimate that the azimuthal fitting precision of the thin filament quasiatomic model is ±6° [37], which for a structure 8 nm in diameter gives a spatial uncertainty of ∼0.4 nm on the perimeter, assuming the actin filament behaves as a rigid body. The expected precision of quasiatomic models is 0.25d where “d” is the resolution [42], or 1.25 nm for 5 nm resolution, which was approximately correct for the myosin lever arms [37].

The location of the head-rod junction of the myosin heads is a particularly important parameter of the model fitting. It was verified by computing separate class averages based on features near the surface of the thick filament backbone, where the lever arms dominate. The raw repeat members of a primary class average were usually distributed over the membership of several thick filament class averages. Several independent fits of the lever arm could then be used to compute an axial and azimuthal standard deviation giving 12.6° or 1.5 nm for the axial orientation and 9° for the azimuthal angle [37]. If there was any doubt about the location of the head-rod junction, we also compared the quasiatomic models built into the primary class averages with the original raw repeats.

Identification of strong and weak binding myosin heads required an explicit criterion. We made this distinction based on the fitting of the MD into the class averages. Strong binding cross-bridges are those for which the MD fit the density without modification from the strong binding configuration, as defined by rigor acto-S1 [5]. Even when the MD was fit without modification, the lever arm usually required significant change. We minimized the amount of axial lever arm change needed for each fit by using two starting atomic structures for strongly bound myosin heads (see Methods), one with the lever arm up, the scallop transition state structure [10], and the other with it down, the chicken skeletal rigor structure [5].

We identify as *weak* binding any cross-bridge that required moving the MD from the strong binding position to fit the density. All apparent weak binding cross-bridge forms were fit from the scallop transition state structure whose MD had been prealigned to the Holmes rigor structure. Although there were other choices for a transition state structure [9], the scallop transition state structure proved to be a good fit to the weak binding bridges requiring little modification and with the lever arm azimuth nearly identical to that of the Holmes et al. structure when their MDs were aligned.

Although it was usually easy to tell whether or not the MD fit the density well without change from the starting structures, once the MD had to be moved the class averages lacked sufficient detail for unrestricted placement. We therefore adopted some guidelines for weak cross-bridge fitting. The entire starting structure was first moved as a single rigid body to get the closest MD fit possible and then the lever arm was adjusted. Lever arm adjustments were both axial and azimuthal. We permitted both azimuthal and axial MD translations and rotations, but usually azimuthal movements sufficed. There was insufficient definition in the MD density to require axial MD tilt to obtain a fit. The MD Cα backbone of weak binding bridges was not permitted to sterically clash with the TM backbone, which was in the high [Ca^2+^] closed position [43]. However, strong binding heads inevitably clashed sterically with TM, consistent with their ability to increase activation of the thin filament by moving TM toward the open position [44].

Two other aspects of the model fitting are important. (1) The classification is not perfect and this affected some classes, in particular the decision as to whether a given cross-bridge is single- or double-headed. Assignment of a bridge class as single- or double-headed was made from examining the class members as described below. A consequence of this is that some single-headed cross-bridge classes contained variable numbers of second heads resulting in average bridge size at a constant contour threshold being larger than that expected for a single head. Likewise, some 2-headed bridge classes had variable numbers of single heads, causing the average 2-headed bridge size to be smaller than expected. (2) The model building was restricted to myosin heads attached to actin. The specimen also contains almost 50% myosin heads not attached to actin, which are located broadly. We expect these to average out, but there is always the possibility that variable amounts of density due to unattached heads will expand the class averages and not be fit by the atomic model.

### Target-Zone Cross-bridge Forms

We expected and found both strong and weak actin attachments in active contraction. There is little previously published information on the structure of weak actin attachments but strong binding attachments are well characterized from combinations of crystallography with cryoEM [5]. Some weak binding attachments with comparatively small MD displacements appeared to be potential precursors of strong attachments since they presented their actin binding interface near to the myosin binding site on actin. Others required much larger MD movements to fit the density. MDA is predicated on the presence of patterns in the data; single occurrences of any kind are effectively noise. Thus, features that occur in the class averages are not chance encounters but are part of a recurring pattern suggesting a consistent feature of actin-myosin interaction.

**Figure 3:**
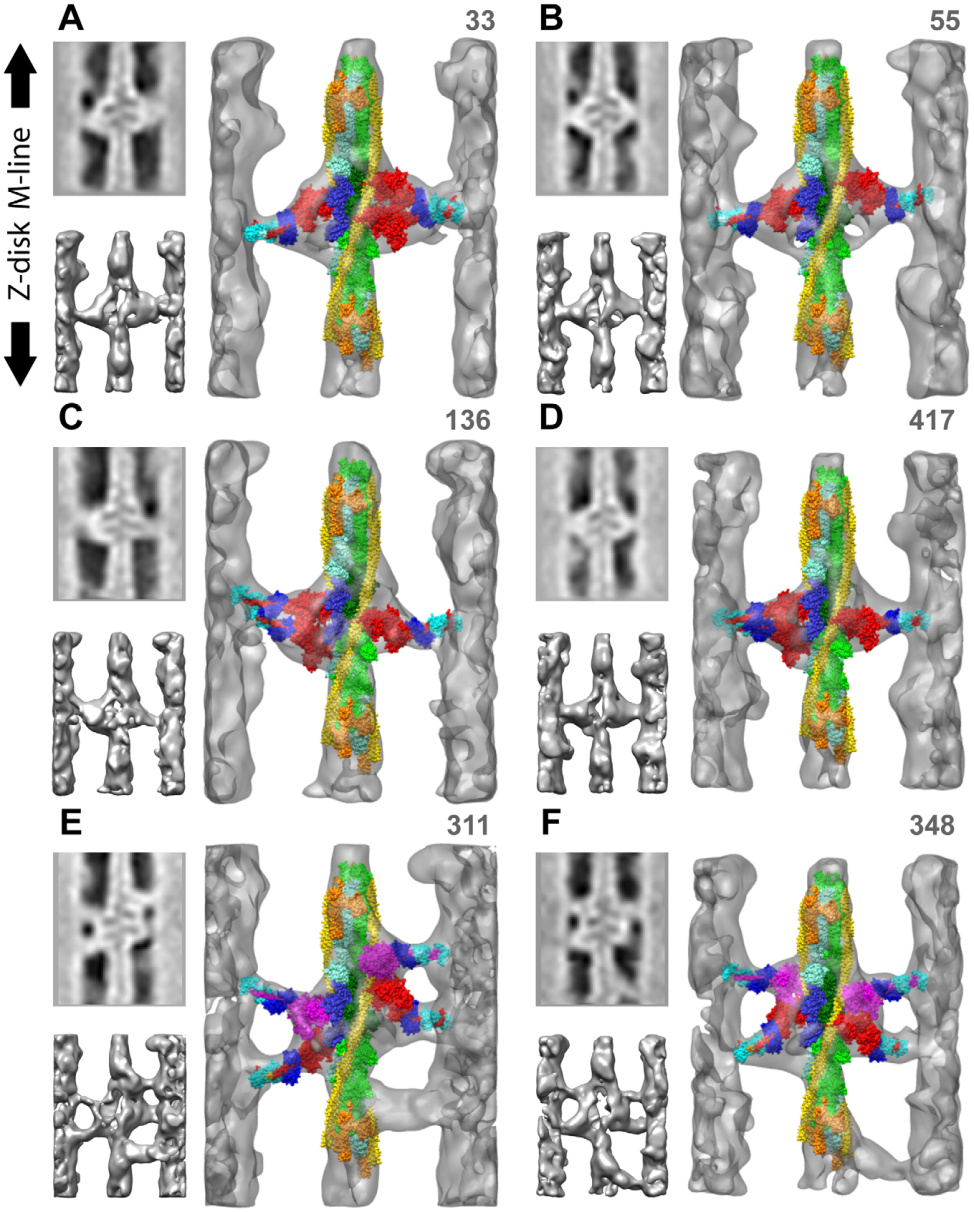
Diversity of myosin-actin attachments shown in quasiatomic models of reassembled primary class averages. The number in the upper right is the number of the corresponding raw repeat, NOT the number of raw repeats averaged within the class. Small panels to the left are the central section and an opaque isodensity surface view of the larger panel without the quasiatomic model. Actin long pitch strands are cyan and green with the target-zone actins in darker shades, TM is yellow and Tn orange. Strongly bound myosin heads are red, weak binding myosin heads are magenta, The essential light chain is dark blue and the regulatory light chain light blue. (A) shows a single headed cross-bridge on the left and a 2-headed, strong binding cross-bridge on the right. (B) shows a pair of 1-headed, strong-binding cross-bridges on actin subunits H and I. (C & D) have a 2-headed cross-bridge on the left and a 1-headed cross-bridge on the right, all strongly bound to actin. (E & F) are mask motifs with Tn-bridges. In (E) the right side M-ward weak binding cross-bridge is bound outside of the target zone to TM near actin subunit F while the one on the left is within the target zone on actin subunit I. In (F), the weak binding, left-side, M-ward cross-bridge is bound outside the target zone to TM near actin subunit G while the weak binding cross-bridge on the right is bound to target-zone actin subunit H. Tn-bridges have not been fit with a myosin head. These six reassembled repeats can also be viewed in Supporting Movies S1, S2, S3, S4, S5 and S6.

We observed six kinds of cross-bridge configurations in the target zone (Figs. 1, 3): mask motifs (Fig. 1A, C, F, M–T, V, W; Fig. 3E, F), 2-headed cross-bridges with both heads strongly bound (Fig. 1D, H, I, K, L; Fig. 3A, C, D), with one strongly and one weakly bound head (Fig. 1G, J), with both weakly bound (Fig. 1I), single headed strong binding (Fig. 1B, D, E, K, L, U, V, X; Fig. 3A, B, D) and single headed weak binding forms of various types (Fig. 1A, J, U, W, X) that are not part of mask motifs. Strongly bound myosin heads were found only on target-zone actins but not all myosin heads attached to the target zone were strong binding.

#### Mask Motifs

Mask motifs are a common structural feature in non-rigor IFM. iso-HST mask motifs were interpreted as consisting of a strong binding head pair on the Z-ward side and a weak binding head pair on the M-ward side [19]. The two heads originate from successive thick filament crowns spaced 14.5 nm apart.

M-ward bridges of mask motifs are positioned on actin subunits F–I (Fig. 3E, F). In the previous work all M-ward members of mask motifs were thought to be precursors of strong binding cross-bridges [19]. However, the present work indicates that strong binding attachments only occur on the four target-zone actins; M-ward bridges on non-target-zone actins F and G apparently must move to a target-zone actin to bind strongly. This they might accomplish by detaching and reattaching as the target zone moves toward them during filament sliding.

#### 2-Headed Cross-bridges

Some class averages appeared to be 2-headed, and when identified, were confirmed by examining galleries of the class members. If the majority of the class members were 2-headed, then the class was identified as 2-headed; if not, then it was identified as single headed. Although 2-headed cross-bridges are common in rigor muscle, they have not been quantified this accurately in active contraction. Two-headed bridges were only found in the target zone. Of the 1528 heads in the target zones, 442 of them (∼29%) were in 2-headed bridges. Of the 221 2-headed cross-bridges, 140 had both heads strongly attached (Fig. 1D, H, I-left side, K, L), 55 had one strongly and one weakly bound head (Fig. 1G, J), and 26 had both heads weakly bound (Fig. 1I - right side).

The 2-headed cross-bridges of rigor usually have one rigor-like head with the second head's lever arm closer to 90° and this was true for one 2-headed cross-bridge class of active contraction, i.e. (Fig. 1I - left side). However, most iso-HST 2-headed cross-bridges do not resemble those of rigor and have both heads with an anti-rigor orientation (Fig. 1D, G–I, L). There is, thus, no rigor-like pattern to the 2-headed cross-bridges of contracting muscle.

### Lever Arm Angle Distribution for Strong Binding Myosin Heads

**Figure 4:**
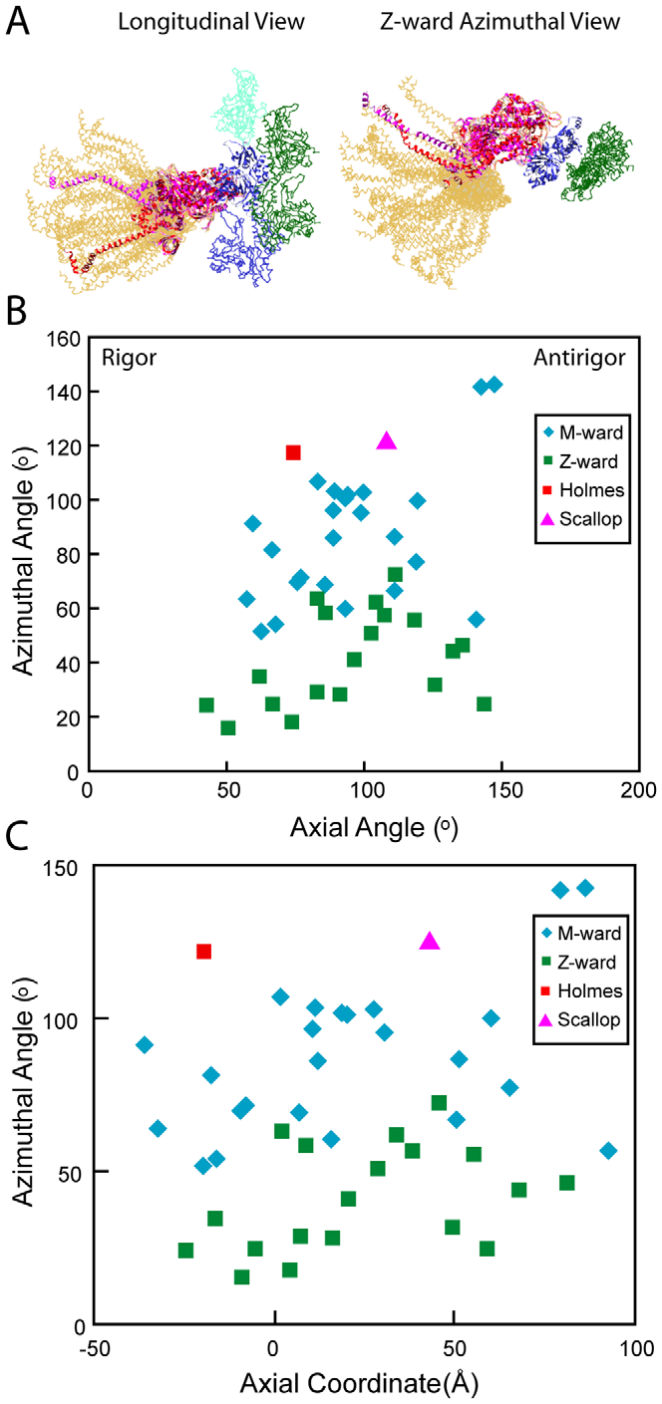
Range of lever arm positions for strongly bound target-zone cross-bridges. (A) Ribbon diagrams are shown for only the heavy chains of all quasiatomic models (gold) and both starting myosin head structures (red and magenta) as docked onto actin in the strong binding configuration. (B) Plot of the axial angle vrs the azimuthal angle for the data shown in (A). Azimuthal angle measured looking M-line toward Z-line. (C) Plot of axial coordinate versus azimuthal angle for the same data. M-ward indicates the values obtained from myosin heads bound to the two actin subunits H and I at the M-ward end of the target zone; Z-ward indicates values obtained from myosin heads bound to Z-ward actin subunits J and K.

**Figure 5:**
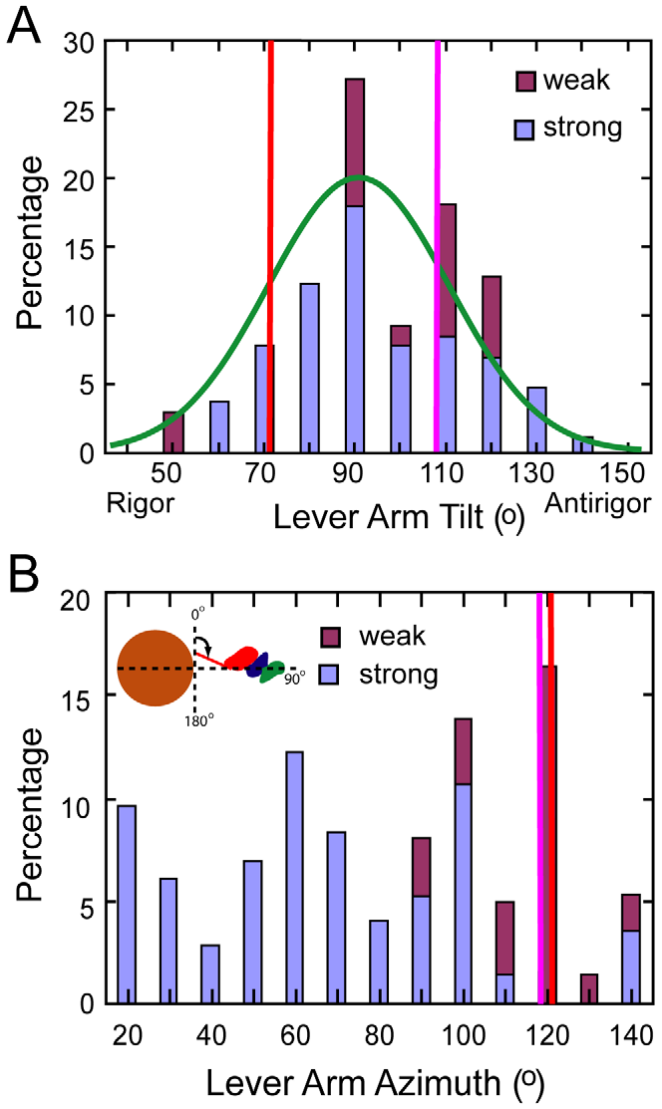
Histogram of lever arm angles for myosin heads bound only to target zone actins. Values obtained after transforming myosin heads to a single actin subunit, I. Weak binding cross-bridges are aligned to the MD of the scallop transition state initial model. Vertical red and magenta lines indicate the position of the initial models within this coordinate frame. The positions of the lever arms for the starting atomic structures are shown as red and magenta vertical lines. (A) Distribution of lever arm tilt angles computed relative to the filament axis. Angles <90° are rigor-like and angles >90° are antirigor-like. The green curve is a Gaussian fit to the data with µ = 95.7°, σ = 19.8°. The fit for strong binding heads alone (not shown) is µ = 93.4°, σ = 19.5°. (B) Distribution of lever arm azimuths relative to the inter-filament axis. The inset gives the angular convention given a direction of view from M-line toward Z-disk. Red and magenta vertical lines show the azimuths of the starting atomic structures, which are very similar. Clearly, if starting-structure azimuth were the only influence, then the final azimuths in B should center around these vertical lines at 120°, as indeed the weak-binding target-zone bridges tend to do here. Direct Z-ward views of the unexpected azimuthal skewing observed for strong-binding bridges are shown in Figure 6B–C, in contrast to the starting-structure azimuths for target-zone myosin heads shown in Figure 6A. Figure 10 depicts possible torsional effects that might contribute to the skewing.

To determine the lever arm axial and azimuthal angles, we used heavy chain residues 707 and 840 in the Holmes et al. S1 structure, or the corresponding residues, 703 and 835, in the scallop transition state structure to define the lever arm axis. The angle between this vector and the thin filament axis defines the axial angle with angles <90° being rigor like and angles >90° being antirigor-like. In this convention, the axial lever arm angle of the Holmes S1 structure is 70.5° and of the scallop transition state structure 107°. When all strong binding heads are transformed to a single actin subunit, the lever arm positions sweep out an arc with an axial range of 77° and a distance of 12.9 nm at the S1–S2 junction (Fig. 4A). These values are more than twice the 36°, 6.4 nm differences between the two starting atomic structures. The distribution is slightly bimodal for strong binding attachments with a shoulder at 110° in addition to the main peak at 90° (Fig. 5A). However, when weak binding attachments are included, the shoulder at 110° is enhanced. We believe this is due to the coupling of lever arm axial angles inherent within paired attachments in mask motifs and in 2-headed bridges. More than half of the 2-headed attachments were strong binding pairs, but nearly all mask motif pairs consist of a strong and a weak binding bridge pair (the left side of Figs. 1M and N are mask motifs with strong binding head pairs).

We determined the azimuthal angle from the projection of the lever arm vector defined above, onto the equatorial plane of the filament lattice. A lever arm azimuth of 90° would be aligned parallel to the inter-thick-filament axis. The azimuthal angles thus defined are dependent on the actin subunit to which the myosin quasiatomic models are transformed, in this case subunit I, but the angular range is not. Surprisingly, the azimuthal range of all strong binding myosin heads is 126° (Figs. 4B, 5B). There is no correlation between axial tilt and azimuth (Figs. 4B, C). The azimuthal angle distribution is bimodal with M-ward bridges being spread over a 52°–143° range (mean 86°±24°) and Z-ward bridges spread over a 16°–73° range (mean 41°±17°). The total spread is unexpectedly wide given that the two starting structures are azimuthally only 4° apart, Holmes rigor, 118°, scallop transition state, 122°. Very few of the fitted strong binding myosin heads have lever arms positioned like the starting structures. The departures are not random but systematic. When viewed Z-ward, nearly all are positioned anticlockwise (with respect to the thin filament) from the starting structures.

**Figure 6:**
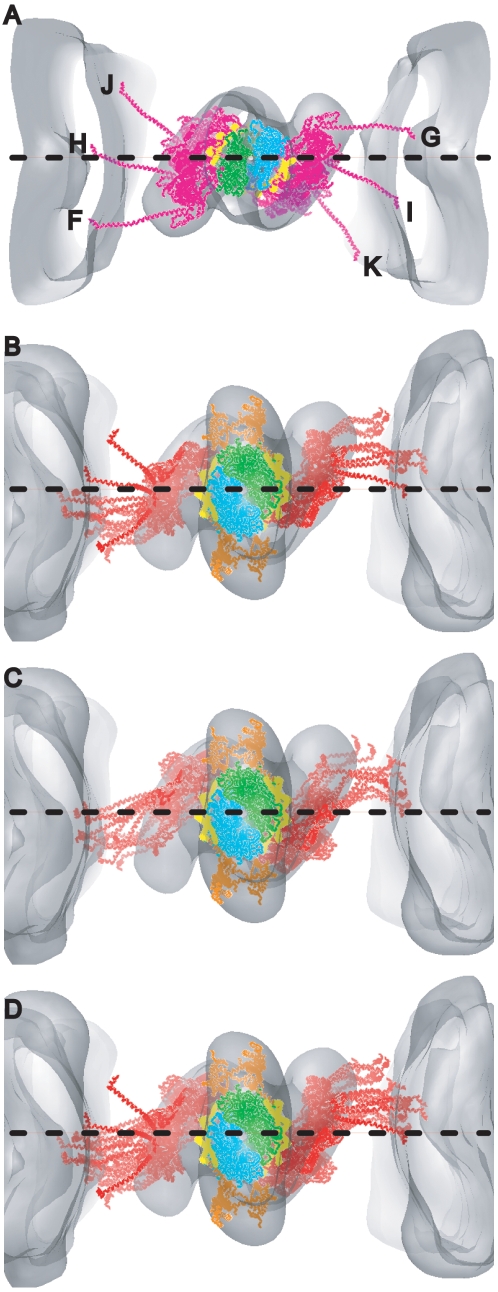
Models of strong binding bridges superimposed and displayed on their bound actin subunits. To provide a spatial reference, the models are displayed with the map of the global average. The horizontal dashed line represents the inter-thick-filament axis. All views are looking from the M-line toward the Z-line. (A) Scallop transition state starting model on actin subunits F–K. The S1–S2 junctions are positioned clockwise from the interfilament axis for all starting models on actins H–K. The S1–S2 junctions of starting models on F and G are located anticlockwise from the interfilament axis. However, no strong binding attachments occur on actins F and G. (B) Bridge models strongly bound to M-ward actin subunits H and I. The lever arms of the only two models that fall above the inter-thick-filament axis are bound to actin subunit H and have the appearance of early beginning-working-stroke conformations. (C) Bridge models strongly bound to Z-ward actin subunits J and K. (D) All strong binding models on their bound actin subunits showing azimuthal distribution skewed notably anti-clockwise from hypothetical dispersions centered around starting model positions in A.

The bimodal azimuthal angular distribution can be attributed to the 26° difference in azimuth presented by the myosin binding site on actin of the two target-zone subunits on each side of the thin filament as a consequence of its helical structure. When the starting structures are placed on the target-zone actins, their S1–S2 junctions are positioned clockwise from the line that connects the centers of the thick and thin filaments (Fig. 6A). The S1–S2 junctions of the myosin heads on the Z-ward actins J and K are positioned further from this line than those on the M-ward actins H and I. The S1–S2 junctions for all but two of the quasiatomic models for strongly bound heads are positioned anticlockwise from this line. The two exceptions are apparently early working-stroke attachments. The range of positions of the S1–S2 junctions at the thick filament surfaces for heads bound strongly to the M- and Z-ward actin subunits almost completely overlap (Fig. 6B–D) despite the 26° difference in azimuth between their bound actin subunits. Thus, cross-bridges strongly bound to either target-zone actin appear as if they originate only from a restricted region of the thick filament independent of two different actin azimuths. Noteworthy is the fact that the S1–S2 junctions of the starting structures placed on actin subunits F and G fall on the same anticlockwise side of the line as the observed strong-binding attachments even though no strong-binding cross-bridges were found on F and G.

Finally, the orientation of the “hook” of the myosin heavy chain in the starting structures (before rebuilding), which connects the myosin head to the S2 domain, is oriented away from the direction that the lever arm has to be bent to fit the strong binding bridges. This would suggest that the forces bending the lever arm azimuthally in situ, might also cause the lever arm to be twisted, a phenomenon which has been observed spectroscopically [45].

**Figure 7:**
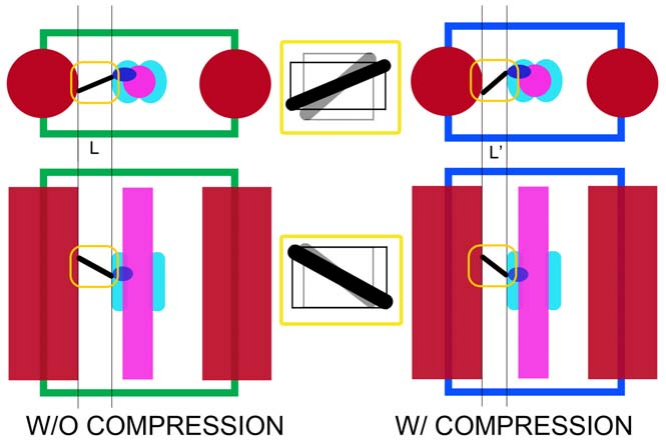
Schematic model of the effect of section compression on the angle of the lever arm. Left illustrates the initial state, prior to sectioning and section compression; right side illustrates the effect of section compression. Top row is the view looking down the filament axis; bottom row is the view looking perpendicular to the filament axis. Color scheme has the thick filament red, thin filament magenta, motor domain blue and lever arm black. The region of the target zone is colored cyan. We assume a worst case scenario, in which the thick and thin filament as well as the myosin motor domain are unaffected by compression and the entire effect is concentrated on the lever arm. Section compression decreases the interfilament spacing with a corresponding increase in section thickness. Widening of the section is assumed to be minimal since the reconstructions are scaled to the axial periodicities. See text for the values obtained from this model.

Section compression, which in the present data reduced the inter-thick-filament spacing from 52 nm to 47 nm, may affect both the axial and azimuthal lever arm angles. We estimated the effect of section compression using a simplified model (Fig. 7). We assumed that the thick and thin filaments are incompressible and that most of the compression occurs in the space between filaments, precisely where the lever arms are located. Section compression of the magnitude observed here would broaden the range of azimuthal angles by ±9°. The effect as modeled would be non-linear as lever arms that were more angled with respect to the inter-filament axis would be affected more. For the axial angle, the effect is smaller, and would broaden the distribution by ±6°. If filaments are compressible so that compression is spread uniformly across all structures, the effect on lever arm angle would be less as the space between filaments is a relatively small proportion of the distance separating the filament centers. Thus, accounting for section compression in the worst case scenario would reduce the azimuthal spread from 127° to 109° and the axial spread from 77° to 65°.

### Weak Binding Cross-bridges

Many weak binding cross-bridge forms were identified in primary class averages, which revealed cross-bridges attached to actin subunits F–K. We divided weak binding bridges into two types, depending on the displacement of their MD center of mass from that of the strong binding attachments, and on whether they contact actin or TM (Table 1, Table S1). Type 1 weak binding cross-bridges had MD displacements of <2.7 nm from the starting structure and their MD contacted the actin subunit; Type 2 attachments were displaced by ≥2.7 nm and their MD contacted TM rather than actin. All Type 1 attachments were within the target zone; five of seven Type 2 attachments were outside of the target zone on actins F and G with the other two on actin H, the most M-ward target-zone actin subunit. MD displacements show a trend from the largest on actin subunits F and G (closest to the M-line), to smallest on actin subunit K (closest to the Z-line).

**Table 1:** Summary of weak attachment models fitted in primary mask class averages.

Repeat #	# of Members	Actin Label	Type	Total Displacement (nm)
298	22	F	2	4.94
343	26	F	2	5.49
311	15	F	2	5.97
343	33	G	2	4.55
348	24	G	2	4.29
126	19	H	2	4.30
107	24	H	2	2.76
117	26	H	1	1.52
348	27	H	1	0.97
246	46	H	1	0.73
356	27	I	1	2.63
73	24	I	1	0.96
311	22	I	1	0.70
126	32	I	1	0.44
246	18	I	1	0.19
117	26	J	1	2.49
118	16	J	1	1.75
356	21	J	1	1.67
*246	46	J	1	1.39
105	34	J	1	0.73
395	21	J	1	0.57
336	23	K	1	0.65

Repeat # refers to the index numbers identified in Figure 1.

# of members refers to the number of raw repeats present in the class.

Actin label refers to the actin subunit given in Figure 2.

Total displacement refers to the displacement of the MD center of mass relative to that of a strongly bound MD placed on actin subunit I. All models were transformed to actin subunit I prior to calculating the displacement.

*Presumed post rigor class average.

**Figure 8:**
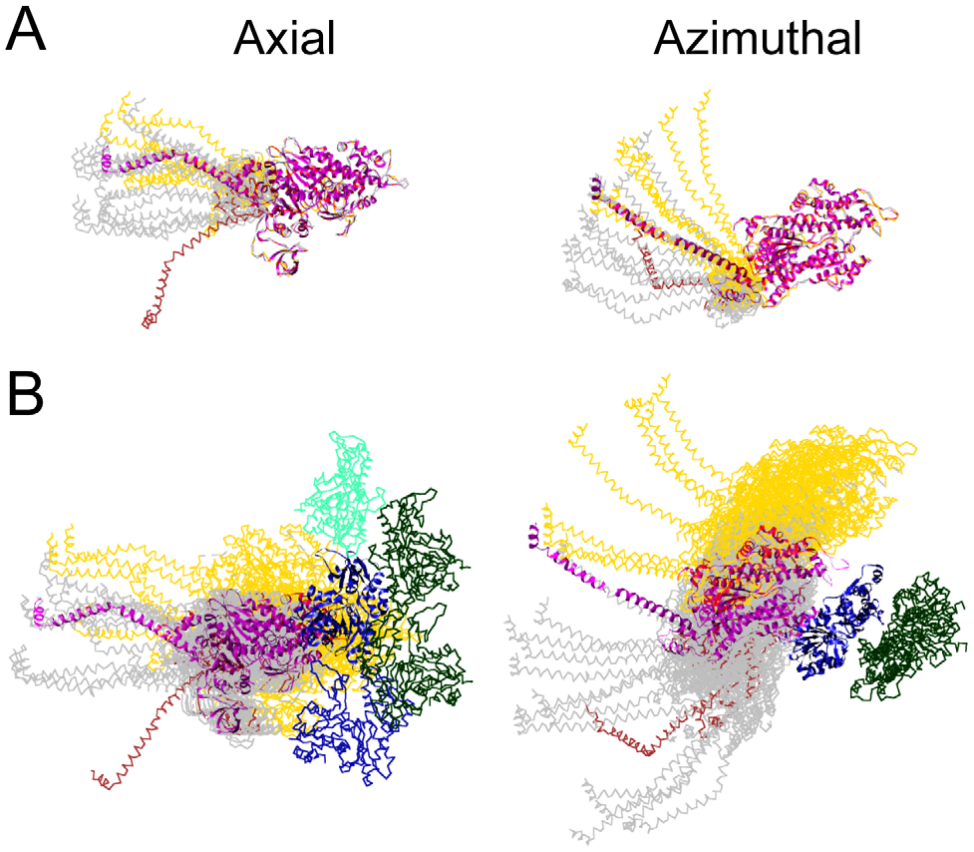
Composite view of weak binding cross-bridge models. (A) Axial and azimuthal views of all weak binding cross-bridges aligned on the motor domain of the scallop transition state structure. This view illustrates the variations in lever arm compared with the starting scallop S1 structure. All weak binding bridges were built starting from the scallop transition state atomic structure, which is shown as a magenta colored ribbon diagram. Type 1 bridges are shown in gray and Type 2 bridges in gold, both rendered as chain traces. The single post-rigor conformation is colored light brown. (B) All weak binding cross-bridges superimposed on actin subunit I. This view illustrates the variations in MD position when referred to a single actin subunit. Coloring scheme is the same as for panel A. Note the relatively small axial dispersion of the Type 1 MDs compared to the broad dispersion of the Type 2 MDs.

We transformed all weak binding cross-bridge quasiatomic models to align their MDs to the starting scallop S1 atomic structure placed on actin subunit I (Fig. 8A) which places them in the same frame of reference as the strong binding cross-bridges described in Figures 4 and 5. One weak binding class, visible in panels W and Q in Figure 1, had a lever arm orientation toward rigor. This class is possibly a post-rigor structure and contained 46 members compared with a total of 443 weak binding bridges. For all other weak binding bridges, the azimuthal angle range is 89° to 142° (Table 2). The ranges for Types 1 and 2 are only partially overlapping, with Type 2 quasiatomic models being distributed to one side of the starting scallop structure (Fig. 8A) and the Type 1 models being distributed on both sides of it.

**Table 2:** Summary of Weak Binding Lever Arm Parameters§.

Crossbridge Type	Number of classes	Axial Angle	Axial Displacement (nm)	Azimuthal Angle
Scallop Transition State	-	107°	+4.50	122°
Holmes Rigor	-	70.5°	−1.86	118°
Type 1	13	92°–122°	+1.76–+6.87	89°–142°
Type 1*	1	53°	−3.8	117°
Type 2	9	88°–93°	+1.03–+7.2	118°–121°
All Weak Binding	23	53°–122°	−3.8–+7.2	89°–142°

§ When MD is aligned to a strong binding MD on actin subunit I.

*Presumed post rigor class average.

Axial displacement measured from the Z-coordinates of the Cα of heavy chain residue 835 of the scallop S1 structure. Axial angle and azimuthal angle measured from the coordinates of the Cα atoms of heavy chain residues 703 and 835.

The average of the axial angles excluding the post-rigor model, 105°±10°, is close to that of the starting scallop transition state model, 107°, and roughly evenly distributed around it. This axial range is less than twice the estimated uncertainty of the quasiatomic models and far less than the range for strong binding heads. Within the confidence limits of the model building, this range represents the inherent flexibility limits between the lever arm and the weakly bound MD.

Type 1 attachments are the most likely candidates for pre-working-stroke forms. When all Type 1 weak cross-bridges are transformed (aligned) onto actin subunit I, as opposed to a strong binding MD on actin subunit I, they suggest a progression in a *clockwise* direction (looking Z-ward) toward the strong binding structure (Fig. 8B). The MD displacements suggested by this alignment are azimuthal in character and would suggest a clockwise azimuthal rotation of the MD (looking Z-ward) to reach the strong binding orientation on actin. A characteristic of the Type 1 cross-bridges is a narrow distribution of MD displacements in the axial direction. A narrow range of lever arm axial angles is present within these models, which is apparently uncorrelated with the azimuthal angles.

When all Type 2 weak binding bridges are aligned to actin subunit I, their MDs are all placed well beyond the azimuthal strong binding position on actin (Fig. 8B) and would require an *anticlockwise* movement (looking Z-ward) along the thin filament to reach a position where strong binding is possible. In contrast to Type 1 weak binding bridges, there is also a significant axial smear of the Type 2 MDs, indicating a significant difference in the ordering of their interaction with the thin filament compared with Type 1 bridges. Thus, the Type 2 weak binding bridges are a new and fundamentally different type of cross-bridge contact (or interaction).

## Discussion

### Comparison with Previous Work

Here we combine modifications in both data collection and analysis to achieve richer and finer detail of the variety of myosin head forms in iso-HST than was possible previously [19]. The improvement is most pronounced in the averages which are important for identifying structural variation in the cross-bridges. Column averages as used previously were only effective at filtering patterns of cross-bridges that repeated axially every 116 nm and is limited in resolution to 12.9 nm, the highest resolution layer line visible in the transform of those tomograms. MDA as used here is effective at identifying different cross-bridge structures regardless of their distribution in the filament lattice. The use of MDA to identify self-similar structures within an ensemble is not limited in resolution by long range order, but is more limited by the number and homogeneity of the structures being averaged.

Previous work utilizing X-ray diffraction and ET concluded that 28–32% of the myosin heads are attached to the target zone for a frequency of ∼2.1 heads/target zone [19]. A subsequent analysis [36] of the same data obtained a binding stoichiometry of 2.6 myosin heads/target zone and 0.52 heads/actin (assuming 5 actins/target) or 0.65 heads/actin (assuming 4 actins/target). The new results indicate 3.0 myosin heads are bound per target zone and averaging 0.75 heads per target-zone actin. The two results are not necessarily incompatible. The earlier results were derived based on two spatial averaging techniques, X-ray diffraction and column averages of the tomogram, both of which observe only repeating patterns in the structure. The present analysis does not require spatial ordering. Irregularly distributed structures, such as the M-ward bridges of mask motifs identified here by MDA would contribute little to X-ray reflections that are enhanced by target-zone bridges.

### Actin Target Zones

The term target zone is defined as the segment of the thin filament where actin subunits are best oriented to form strong attachments to myosin heads projecting from adjacent, parallel thick filaments. Though defined initially for IFM [35], the concept is entirely general not only for organized filament arrays such as striated muscle [33], [34], [46], [47] but also for *in vitro* single-molecule experiments using various myosin isoforms [48]–[53]. However, up to now the target-zone size has not been so precisely defined.

Previously, we inferred that the target zone was on average 2.5 actins long on each long pitch helical strand [19], [36], three actins on one strand and two on the other, alternating sides with successive crossovers. The present result, in which actin subunits and Tn's are resolved, and weak and strong binding attachments are distinguished, shows that the target zone comprises just two subunits on each actin strand, positioned exactly midway between two successive Tn's on that strand. The frequency of all myosin attachments increases 10 to 30-fold on target-zone actins relative to the nearest neighbors. Moreover, strong binding heads disappear abruptly outside of the target zone. The lack of any strong binding cross-bridges on actin subunits F and G, excludes these actins from the target zone where previously they were included [36].

We think that the highly restricted target zone cannot be due to the assumptions used to distinguish strong and weak binding attachments. Only Type 2 cross-bridges are found on M-ward actins F and G; even Type 1 attachments are not found on these actin subunits. Moreover, virtually no attachments of any kind were found on Z-ward actins L and M.

The relative orientation of actin subunits with respect to myosin head origins is the most obvious factor defining the target zone [35], [54] but the size of the target zone can vary. In rigor the target zone encompasses eight successive actin subunits along the thin filament genetic helix (actin subunits H–O), i.e. four on each long pitch strand. Two-headed “lead” cross-bridges (leading toward the M-line) occupy the four M-ward subunits (H–K) and the usually single-headed “rear” cross-bridges are bound to the most Z-ward pair (N, O) [55]. The extensive deformation of rigor rear bridges and their complete absence in AMPPNP [27], [56], suggests that the high actin affinity of nucleotide-free myosin extends the limit of acceptable actin azimuth.

Our sharply defined target zone implies a restrictive geometrical constraint on myosin head binding that is contradicted by the highly variable shape of strong binding cross-bridges, in particular their widely varying azimuthal lever arm orientations. If our two extremes for lever arm azimuth of strong binding myosin heads reflected intrinsic, state independent, myosin head flexibility, some heads should be able to bind strongly at all but two actins, D and E, while staying connected to the thick filament. Moreover, if actin azimuth alone were the limiting factor, we would expect a more gradual tapering of strong binding attachments from the center of the target zone. This we also do not see.

Type 1 weak binding attachments show a smaller azimuthal lever arm variation (Figs. 4A, 5B) than the strong binding heads that they evolve toward. However, when the two azimuthal lever arm extremes of Type 1 weak actin attachments are transformed to the position of strong binding motor domains on each actin subunit, some heads should be able to bind strongly at all but four actins, D, E, R, & S, while staying connected to the thick filament. The initial myosin crystal structures sit near one azimuthal extreme of our strong binding attachments (Fig. 5B). When placed on actin in the strong binding configuration, simultaneous myosin-actin attachments can be made only at six actins F–K (Fig. 6A), which includes the entire target zone. It is notable that the lever arm azimuth in this case would exclude actin subunits L and M from strong myosin attachments but our results show that even non-specific, weak attachments are exceptionally low on those subunits.

Based on the two initial crystal structures, we would expect that strong binding bridges would be found on actins F and G, but none are found. Even Type 1 weak binding bridges are not found on actins F and G. This suggests that an additional factor is limiting the target zone which may be the dynamic properties of TM. The Tn complex adds additional actin affinity to TM and holds it relatively securely at the ends of the actin repeat period but motion of TM would be expected to be highest midway between Tn complexes, exactly where the target zone is located. Reconstructions of actin-TM-Tn done by single particle methods revealed the weakest TM density midway between Tn complexes [57] consistent with the idea of high mobility in this region which coincides with the IFM target zone.

### Distribution of Actin-Bound Myosin Heads

Our results show that myosin head attachments occur all along the thin filament, although with much lower frequency than the target-zone attachments. Target-zone attachments account for 78% of all attachments but utilize only 28% of the actin subunits. The other 22% of attachments are distributed among the remaining 72% of actin subunits. Many of these attachments are non-specific with respect to the myosin binding site on actin. Some may represent collision complexes, but others, such as the Type 2 weak binding bridges on actin subunits F and G are both numerous and have a well defined appearance in the averages suggestive of some kind of specific interaction, even if novel. Outside of the target zone, actin subunits are labelled only 8% of the time on average (range of 3% to 13%). Nevertheless, these non-specific attachments occur with enhanced frequency on the 4 actin subunits near Tn and with strikingly low frequency on two actin subunits (L and M) adjacent to the Z-ward side of the target zone.

Initially, we thought actin subunits L and M never had myosin attachments, but a classification designed specifically for this region found some attachments, the density of which was generally poorly defined. Although it is possible that this represents statistical uncertainty, it may also indicate something special about these actin subunits. Non-specific attachments occur everywhere outside the target zone so the especially low frequency here may indicate an adaptation for IFM.

One in every seven subunits of IFM thin filaments is a ubiquinated form of actin named arthrin [58], [59]. Although arthrin is regularly spaced every 38.7 nm along *Drosophila* IFM thin filaments [60], its location is close to Tn, and not to the target zone [61]. Thus, arthin located at positions L and M is an unlikely explanation.

### Limitations on Cross-bridge Attachment

**Figure 9:**
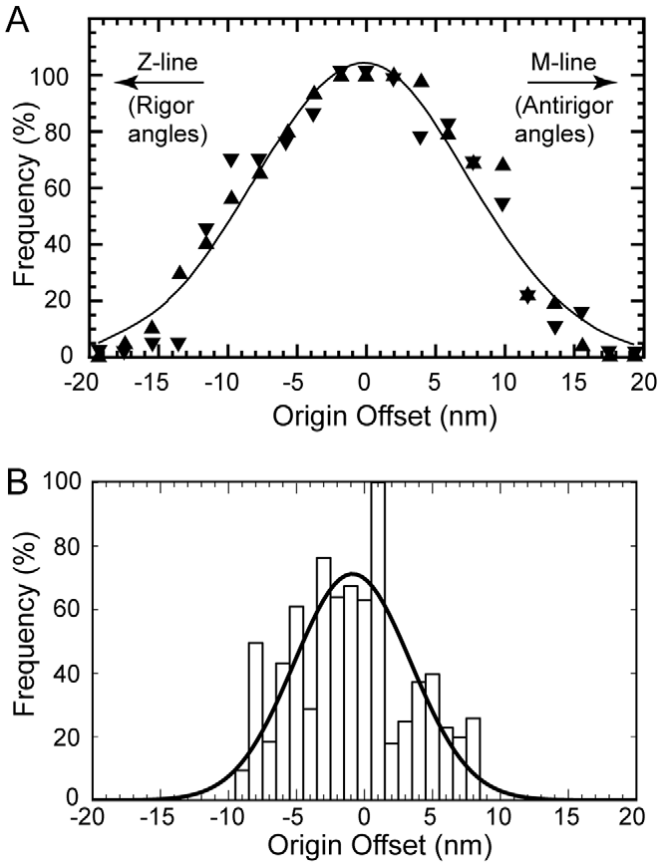
Probability of target-zone cross-bridge formation as a function of cross-bridge origin. (A) Data reproduced from Tregear et al. (2004) in which the target zone is assumed to be three actin subunits on one side and two actin subunits on the other. (B) Data from the present study, which includes only target-zone cross-bridges on two actin subunits from each side. Although the Tregear et al. data, which were measured by hand, have an overall Gaussian shape, the present measurements, which are based on quasiatomic model fitting, do not follow a strictly Gaussian distribution. The continuous line is a Gaussian fit with µ −0.86 nm and σ = 4.3 nm.

Tregear et al. [36] reanalyzed the earlier data [19] combining column averaged images to define target-zone locations and cross-bridge origins on the thick filament with the raw tomogram to determine the angles and positions of all individual attached cross-bridges. Tregear et al. concluded that the binding probability of cross-bridges to the target zone followed a Gaussian distribution that depended on the axial offset between target-zone center and the shelf of cross-bridge origin (Fig. 9A). Their methods could not discriminate between weak- and strong-binding attachments in and around the target zone and thus deemed the target zone to be larger than found here.

For comparison, we have replotted our fitting data shown in Fig. 9B by referring the axial coordinate of all lever arm C-termini (P840 for Holmes and P835 for scallop) to the center of the target zone. Data from left and right hand sides of the target zone are combined, but referred to the center of the individual side, not the overall center of both. The shape of the resulting distribution is not Gaussian but when fit to a Gaussian, the average is −0.84 nm (angling towards rigor). All of the measured points fall within ±10 nm of the target-zone center and 84% fall within ±5.5 nm. If the present data are referred to the overall center of the paired target zones making it comparable to Tregear et al., the distribution would be spread an additional 1.35 nm on either side which would mean that all data fall within ±12.7 nm. For the Tregear et al. data, ∼85% of all attached cross-bridges originate within ±11.4 nm of the center of the target zone. The present analysis is therefore broadly consistent with Tregear et al. and differs primarily in its details that are better defined due to the higher resolution of the reconstruction.

### The Asymmetry of Actin Attachments

Our results show that the number of actin-myosin attachments is relatively symmetric within the target zone, but is very asymmetric just outside the target zone, where the asymmetry in numbers is matched by an asymmetry in structure. The two actins M-ward of the target zone (F and G) are comparatively well occupied, while attachments to actins L and M on the Z-ward side of the target zone are rare. Weak attachments on actin subunits F and G (Type 2) differ the most from strong binding attachments, while those on actin subunit K (Type 1) have the smallest MD displacements from strong binding and are fewer in number.

Muscles are designed to generate tension while shortening and accomplish this by using filaments that are polar within the half sarcomere. Thus, the asymmetry we observe is not unexpected given the filament structures themselves. When a muscle shortens the target zone on the actin filament moves toward the M-line. On the M-line side of the target zone, on actin subunits F–H, we find the most unusual of the weak binding cross-bridge forms, Type 2. Although considered weak attachments, their structure is well defined in the averages and they are the only attachments found on those actin subunits. While not positioned on actin in a way that readily converts to strong binding, Type 2 attachments are placed so that if they remained stationary while the actin target zone moved M-ward by 1 or 2 actin subunits, they would be positioned to “bushwhack” the target-zone actins and form Type 1 weak attachments (Supporting Movie S7). If the movement were larger, we can identify no weak attachments that would be in a position to quickly bind the target zone.

Conversely, if the muscle is stretched, which is to say the target zone moves Z-ward, there are literally no myosin heads positioned to bind the target zone. Adaptation to stretch would therefore require a different mechanism for rapidly placing more myosin heads on target-zone actins. It has been suggested that when a muscle is stretched, two-headed attachments increase by recruitment of second heads to single-headed bridges [62]. Our results show that two-headed cross-bridges exist in isometrically contracting muscle even without stretch which shows directly that this mechanism for adapting to stretch is mechanically feasible in active IFM.

The two types of weak binding attachments identified here may play differing roles in the recovery of tension after a quick release. Type 1 attachments are closest to the strong binding configuration and would thus appear to be best suited for contributing to Phase 2 rapid force recovery if the release distance was ∼5 nm [63], [64]. Lombardi et al. [65] observed that the stiffness attributable to attached bridges was constant up through Phase 2, while Bershitsky et al. [22] found that stiffness of weak binding bridges that were not stereospecifically attached to actin was unchanged during the weak-to-strong transition that brought about stereospecific attachment and force development. Therefore the Type 1 bridges seen here that are not stereospecifically attached could well be capable of contributing to the stiffness attributed by Bershitsky et al. to weak binding bridges, yet transform without stiffness change to stereospecific strong binding force generating bridges during the phase 2 rapid force recovery observed by Lombardi et al. The Type 2 attachments, which are not attached to actin at all but rather contact TM, may instead be responsible for a more delayed Phase 3 tension recovery.

What exactly is holding the Type 2 bridges in place is not visible in the reconstructions. Their position is variable with respect to TM, both azimuthally and axially which argues against a specific interaction. One possibility is the N-terminal extension of the regulatory light chain. The sequence of this extension is known in *Drosophila*, which has led to the suggestion that it positions myosin heads for attachment to the thin filament [66]. Its validity for *Lethocerus* will depend on the demonstration of a similar N-terminal extension.

### 2-Headed Cross-Bridges Are Not Rare in Isometric Contraction

Based on column averages, it was concluded that the great majority of cross-bridge attachments were single-headed [19], and a subsequent analysis of the same iso-HST tomograms drew the same conclusion [36]. The present analysis indicates that 2-headed attachments are in the minority, but are not rare. Approximately 14% of the cross-bridges (all in the target zone) contain both heads of one myosin molecule. For the single-headed cross-bridges, we believe the companion head is either mobile or remains close to the relaxed state docking position on the thick filament shaft producing the residual density of the myosin shelves spaced 14.5 nm apart. These shelves are prominent in iso-HST but not in rigor where 2-headed binding predominates [55].

Single headed myosin attachments are consistent with a variety of other structural evidence such as X-ray modelling of relaxed insect thick filaments [67] and various experiments on vertebrate muscle, both in situ [32], [68], [69] and in vitro [70], [71]. Conibear et al. have argued on energetic grounds that 2-headed binding is unlikely during active cycling of myosin [44]. However, 2-headed attachments are consistent with several sets of experiments involving single molecule motility [72]. The present result indicates that at least in isometric contraction, two-headed myosin attachments are significant but are not the major cross-bridge form.

### The two stage working stroke

Previous analysis of iso-HST tomograms elucidated a working-stroke sequence by arranging all the quasiatomic models of cross-bridge fittings in a sequence based on the coordinate of the C-terminal residue at the S1–S2 junction [19]. The sequencing suggested that the working stroke consisted of two stages, one in which both the MD and the lever arm move from an M-ward tilt toward a Z-ward tilt in Stage 1, with Stage 2 beginning when the MD settled into the strong binding configuration and only the lever arm moved. The present results are not easily compared with that work for several reasons. (1) Cross-bridge features seen earlier had to be spatially repetitive; averages would be computed over heterogeneous structures if this were not true. The present result is not so limited and averages have been computed over structures that are more homogeneous even though irregularly placed in the filament lattice. (2) The independent fitting of a thin filament quasiatomic model into the present maps provides an improved criterion for distinguishing weak from strong binding cross-bridges. (3) The greater detail of the weak binding target-zone bridges and the availability of two crystal structures, one a transition state with the lever arm elevated and the other a rigor structure, reduced the amount by which the starting models had to be altered to fit the density. Thus, previously the motor domain placement of weak binding cross-bridges involved both axial and azimuthal changes in the MD, but the present fittings of weak binding bridges could be fit using only azimuthal MD changes.

In the sequential ordering of structures from weak-to-strong binding [19], the azimuthal range of lever arm positions narrowed as the lever arm moved from the initial stage to the final, rigor-like stage of the working stroke. The present work differs in that a large azimuthal range of the lever arms persists at all stages. Thus, the present work does not support the conclusion that the azimuthal distribution of the lever arms is wide in the weak binding states and narrows as the myosin heads go through the working stroke. The distribution of lever arm azimuths is widest for strongly bound heads and smaller for weakly bound heads. However, the number of different weak binding forms does suggest new details of the transition from weak-to-strong binding.

#### Stage 1- weak attachments

We characterized two types of weak attachments between myosin heads and the thin filament, with Type 1 attachments being closest in appearance to strong binding cross-bridges and with Type 2 being decidedly different. We think that Type 1 could be pre-working-stroke attachments because their MD position is closest to the strong binding configuration and they occur only within the target zone. Type 1 weak attachments are consistent with the idea that initial binding need not be precise [73] but rather is disordered as observed by cryoEM [74], Electron Paramagnetic Resonance [23], and X-ray fibre diffraction [22]. Random thermal motions would then align the MD into the strong binding configuration possibly generating force [75].

Our class averages at the moment lack the resolution and signal-to-noise ratio to be unambiguous but they suggest the following properties of the weak binding attachment that precedes strong binding. Type 1 cross-bridges can be arranged into a weak-to-strong binding sequence (Fig. 8B), but this would embody mostly azimuthal movements of the MD across the actin surface, toward TM and the strong myosin binding site on actin. The present data lack the resolution to exclude small changes in MD tilt as part of the transition. In this sequence, MD progress toward the strong binding site on actin displays a corresponding but uncorrelated set of comparatively small axial and azimuthal lever arm movements.

With the exception of one Type 1 weak binding cross-bridge, a possible post rigor conformation, all the weak binding forms on actin subunits F–K display a narrow range of axial and azimuthal lever arm orientations when aligned to the MD (Figs. 5B, 8A). The azimuthal lever arm range of the weak binding bridges is much smaller than the range found for the strong binding bridges, and is rather symmetric with respect to the lever arm position of the crystal structures consistent with inherent flexibility. Conversely, for strong binding cross-bridges, the distribution is not only large, but is strongly biased toward one direction, suggestive of a genuine characteristic of isometric force generation in situ.

#### Stage 2 strong binding

The fitting of strong binding cross-bridges generally required considerable axial alteration of the lever arm for both crystal structures, which differ by 36°/6.4 nm compared with the 77°/12.9 nm range observed here. Our observed range is consistent with previous estimates made by comparing crystal structures of myosin catalytic intermediates from different isoforms [8], [10] and is also similar to that observed earlier [19]. Section compression as modelled here could broaden the spread by about ±6° leaving a maximum working stroke of ∼10 nm. Despite these factors, our range is smaller than the 105° observed by comparison of reconstructions obtained from actin filaments decorated in AMPPNP or ADP with a myosin V construct containing two IQ motifs with bound calmodulin [15].

The average axial lever arm angle obtained from a Gaussian fit to the data in Figure 5, gave a mean angle of 93°±20° for the strong binding cross-bridges, nearly perpendicular to the filament axis and in good agreement with values that were found to fit the meridional X-ray reflection from isometrically contracting vertebrate muscle fibers [76], [77].

A consistent and surprising feature of the strong binding cross-bridges is the large, asymmetric distribution of the lever arm azimuths relative to the starting models. This is a feature of the data that has been extensively checked against different applications of MDA as well as against the original raw tomograms so we believe it to be real. The width of the distribution is affected by section compression, but even accounting for an additional 9° at either extreme, still gives a range of 110°. These azimuthal lever arm changes relative to the two initial structures gives the cross-bridges a more straightened appearance.

Cross-bridges with a straightened appearance have been observed previously in situ. They were first reported in EMs of IFM following AMPPNP treatment [78], and in EMs of quick-frozen, contracting vertebrate muscle [32], [33]. IFM reconstructions of rigor [25], [26] and following AMPPNP treatment [27], [28], [56] also show this effect. Thus, we think that this feature of the cross-bridge structure is not unprecedented. In the previous IFM work, it was uncertain whether the observation was an isoform difference or was revealing a novel feature of in situ cross-bridges, and there was no control group in the reconstructions in the form of unattached or weakly attached heads for comparison. That this observation is not a species difference is supported by the recent 3-D reconstruction of actin filaments decorated with IFM myosin S1, which have some small differences, but largely are very similar to rigor acto-S1 from other isoforms of myosin II [79]. The weak binding bridges in the present reconstruction are a control group. Their smaller and more symmetrical distribution about the initial models suggests that for weak binding bridges we are observing flexibility and for strong binding bridges we are observing an aspect of the working stroke of myosin heads functioning in situ.

### Correlations with X-ray Fiber Diffraction

Correlated changes in the actin layer line intensities with characteristic myosin reflections using an applied jump in temperature during isometric contraction [21], [22] show that tension increased with the rise in temperature indicating an increase in strong binding cross-bridges. However, fiber stiffness and the intensity of the 1,1 equatorial reflection did not change, indicating constancy of numbers of actin attached cross-bridges. Moreover, the intensity of the 38.7 nm layer line, which measures stereospecific attachment to actin rose, while the intensity of the 1,0 reflection, which measures mass ordered on the thick filament decreased. These changes were interpreted to indicate that the structure of attached cross-bridges changed and that these changes are largely azimuthal with respect to both thick and thin filaments. An increase in stereospecific actin-myosin attachments would increase the 38.7 nm actin layer line as observed, but cannot be accounted for if the myosin head attaches weakly to actin in the stereospecific orientation in which case strong binding simply closes the actin binding cleft. The observed increase requires a more dramatic change in disposition of the MD on actin during the weak-to-strong transition, as observed here.

In vertebrate striated muscle, the intensity ratio of the 1,0 and 1,1 equatorial reflections is a measure of the change in myosin heads moving from thick filaments to thin filaments and these changes normally correlate with tension generation in isometric contractions [80], [81]. Equatorial X-ray diagrams at low ionic strength (µ = 50 mM), which enhances the population of weakly attached cross-bridges even in the relaxed state to nearly 80% of those available, revealed little change in the 1,1 equatorial reflection but a large decrease in the 1,0 equatorial intensity when Ca^2+^ activated, indicative of movement of mass away from the thick filament [82]. This observation was attributed to a pronounced structural change in the already attached cross-bridges; this could not be explained if the MD of weak binding bridges were already oriented as in rigor but with the actin binding cleft open, needing only to be closed for strong binding, a structural change too limited to account for the X-ray change.

### Meaning of the Azimuthal Changes in Strong Binding Cross-bridges

Accepting that our skewed azimuthal lever arm distribution is a property of active cross-bridges, the obvious question is what does it mean for myosin function in situ? Is it a reflection of intrinsic myosin head flexibility, is it an aspect of the weak-to-strong transition, or is it an aspect of the myosin working stroke? The broad lever arm azimuthal angular range seen for strong binding bridges is a conundrum. Placement of strong binding cross-bridges on actin can be limited only if the myosin head is given limited flexibility as suggested by the two crystal structures used for the fitting. Yet the changes observed in the strong (and weak) binding cross-bridges compared with the crystal structures imply substantial azimuthal flexibility that, if intrinsic to myosin heads, would permit strong binding bridges to form virtually anywhere along the actin 38.7 nm repeat. Even in rigor, where actin affinity might increase the target-zone size, cross-bridges are still largely confined to the target zone of contracting muscle. Intrinsic myosin head flexibility would seem to be an insufficient explanation for the strongly biased azimuthal change.

If the azimuthal lever arm distribution for strong binding cross-bridges were an aspect of the working stroke, we might expect to see a relationship between axial angle, representing progress through the working stroke, with increasing, or decreasing azimuthal angle. However, graphs of axial tilt angle versus azimuthal angle for the strong binding cross-bridges fail to show the obvious correlation (Fig. 4B, C) expected if the two motions were coupled.

That there is no coupling between axial and azimuthal lever arm angles may be an effect of S2. Cross-bridges do not emerge from the thick filament backbone at the S1–S2 junction; they originate where S2 emerges from the thick filament backbone. S2 is widely thought to provide a flexible tether that can bend radially, as well as azimuthally around the thick filament surface, to facilitate actin-myosin attachment. If S2 swings azimuthally during cross-bridge attachment so that it becomes angled with respect to the filament axis when force is initiated *down* the filament axis, that force will have both an azimuthal component (a torque) as well an axial component. If the S2 swing is anticlockwise (looking Z-wards), then the angled S2 would produce a torque that bends the lever arm clockwise with respect to the thick filament. In this case, the torque would straighten the myosin head compared with the starting crystal structures, in accord with our observation. If the S2 swing was in the opposite direction (clockwise), the lever arm would bend anticlockwise with respect to the thick filament thereby making the myosin head more bent than the already bent S1 crystal structures contrary to our observation.

In iso-HST, we do not observe where S2 emerges from the thick filament backbone, we only see the S1–S2 junction of the lever arm and so cannot directly evaluate this effect. Where S2 has been observed in swollen IFM fibers, the range of directions suggests that S2 swings equally well both clockwise and anticlockwise, about the thick filament surface [26]. A symmetrical distribution of angles for S2 with respect to the filament axis at the beginning of force production might explain the lack of coupling between axial and azimuthal lever arm angles of strong binding bridges, but it does not explain the strong directional bias (Fig. 4).

**Figure 10:**
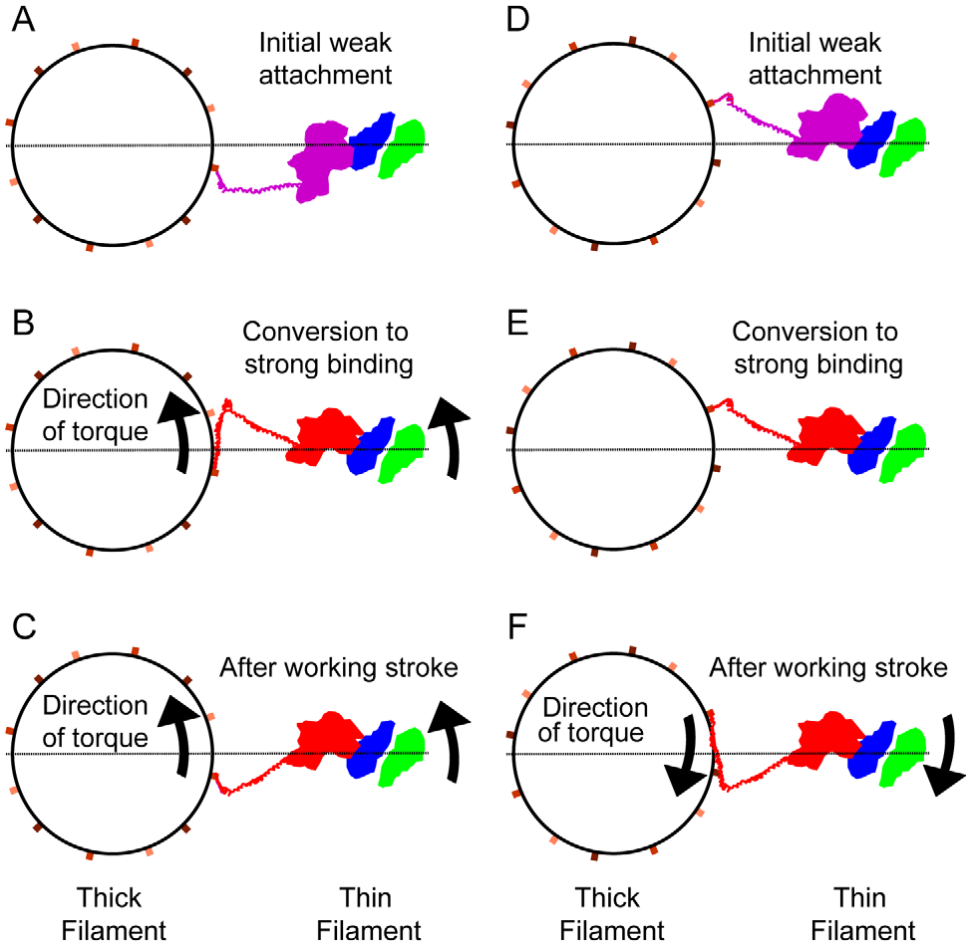
Two mechanisms to account for azimuthal skewing of lever arms of strongly bound cross-bridges. (A–C) Conversion from weak-to-strong binding according to Scenario 1. (D–F) Active azimuthal component to the working stroke. View direction is M-line toward Z-line. Myosin is colored either red (strong binding) or magenta (weak binding). Actin subunits are green and blue. Three successive levels of S2 origins are shown in shades of brown that darken with distance from the observer. The lever arm is the line originating on the red (or magenta) MD while S2 is shown as a short segment when oriented nearly parallel with the filament axis and becomes longer when angled with respect to the filament axis. The horizontal line is the inter-filament axis. Arrows show the direction of the torques (not their magnitude) produced during the weak-to-strong transition or as a component of force generation and filament sliding. The direction of thin filament movement during sarcomere shortening is toward the observer. (A & D) Initial weak binding is shown, which in (A) begins away from the strong binding orientation and in (D) begins in the strong binding orientation but with actin binding cleft open. (B) Conversion to strong binding involves diffusion of the MD clockwise on actin which swings the S2 anticlockwise about the thick filament. (C) Force production realigns S2 with the filament axis while bending the lever arm azimuthally. (E) Transition from weak to strong binding involves no change in myosin orientation on actin, just a closing of the actin binding cleft. The lever arm in (D & E) is in the same orientation suggested by the crystal structures. (F) An azimuthal component to the working stroke moves the lever arm clockwise around the thick filament. This figure can be seen as an animated sequence in Supporting File S1.

We can think of two mechanisms that would bias the azimuthal bend of the lever arm to produce a cross-bridge that appears azimuthally straightened in comparison to the crystal structures. One of these involves the weak-to-strong transition, the other invokes an active azimuthal component to the working stroke. Either mechanism would enable the S1–S2 junction to be positioned anticlockwise of the inter-filament axis as depicted in Figures 6 and 10.

#### The weak-to-strong transition

The range of structures of Type 1 cross-bridges (Fig. 8B) suggests that the weak-to-strong transition involves a concerted azimuthal translation and rotation of the MD in a clockwise direction (looking Z-wards) about the thin filament (this direction is also toward TM). Two scenarios that involve different compliance between the lever arm and the S2 may serve to illustrate.

In the first scenario (Supporting Movie S8, Fig. 10A–C, Fig. S1), the myosin head is assumed initially noncompliant and so does not change as it moves azimuthally across the target-zone actin during the weak-to-strong binding transition. Instead, S2 is assumed compliant and begins this transition aligned parallel with the filament axis (Fig. 10A), but the myosin head movement swings S2 azimuthally (anticlockwise about the thick filament looking Z-ward) angling it with respect to the filament axis (Fig. 10B). When the working stroke applies axial force to the lever arm, the angled S2 will produce a torque that will pull the lever arm clockwise with respect to the thick filament as S2 aligns with the filament axis (Fig. 10C). The myosin head at the end has become straightened.

In the second scenario (Supporting Movie S9; Fig. 10A,C), S2 is non-compliant and so resists being swung azimuthally during the weak-to-strong transition. Instead, the lever arm of the myosin head is compliant and bends azimuthally clockwise (looking Z-wards) with respect to the thick filament during the transition. Essentially, the transition would go from Figure 10A to Figure 10C, bypassing the state depicted in Figure 10B. When strong binding occurs and the working stroke is initiated the force is applied axially with a minimal torque but the lever arm is already bent azimuthally.

The first scenario is supported by the two strong binding structures which fell above the inter-filament axis in Fig. 6B and could be interpreted as very early working-stroke attachments. The second scenario is supported by the lever arm azimuthal distribution of Type 1 weak binding bridges which are somewhat straightened azimuthally, but the amount is small compared to strong binding cross-bridges. Thus, there are structures within the ensemble to support either scenario.

The first scenario bears some resemblance to the “roll and lock” mechanism described by Ferenczi et al. [83]. This detailed model incorporates the concept that myosin in kinetic states with ATP or ADP•Pi bound make variable attachments to actin that differ not only in the azimuthal position on actin, as suggested by the present data, but also with variations in axial orientation as suggested by earlier work on the I-HST state of IFM [19]. The present work shows highly variable attachments all along the thin filament, but only weak attachments within the target zone (Type 1) are likely candidates to transition to strong binding. The variation in their orientation on actin of this group is much smaller than envisioned in “roll and lock”. In addition, the present work does not require MD tilt in the Type 1 weak attachments, but we think the data at the moment do not definitively rule out some MD tilt as part of the weak to strong transition. Whether Type 2 attachments can “roll” on actin is doubtful because their MDs do not appear to attach actin at all. Nevertheless, the present data support at least some aspects of the “roll and lock” model for the weak to strong transition.

#### An azimuthal component to the working stroke

Several observations made with in vitro motility assays have shown a torque component to the working stroke, dubbed “twirling”, inferred from rotations of the actin filament in gliding assays [84], [85]. Twirling as generally observed with myosin II involves a left-handed actin filament rotation during filament sliding. With an active azimuthal component to the working stroke, azimuthal diffusion in the weak-to-strong transition described above is not required. Initial weak binding could place the myosin head on actin oriented as in strong binding but with the actin binding cleft opened (Fig. 10D) and the lever arm azimuth positioned as in the crystal structures. The weak-to-strong transition would simply involve closure of the actin binding cleft without a change in orientation of the MD (Fig. 10E). If the working stroke involved an inherent clockwise rotation of the lever arm about the thick filament when looking Z-wards, the S1–S2 junction would move clockwise about the thick filament (Fig. 10F) and the cross-bridge would be straightened. A consequence of this mechanism is that the myosin origin (i.e. where S2 emerges from the thick filament backbone) could be positioned initially above the inter-filament axis (Fig. 10D), where the crystal structures predict it should be, and the S1–S2 junction would, after tension developed, appear below the inter-filament axis where we observe it in situ.

The azimuthal movement of the lever arm under this mechanism would not only straighten the cross-bridge but would also impose a clockwise torque on both the thick and thin filament, opposite the one imposed by the weak-to-strong transition. If filament sliding was possible, the applied torque would impose a left handed rotation to the thin filament, matching the observation in vitro [84]. For myosin II, Beausang et al. observed a screw pitch of 470 nm, about half the length of thin filament observed in our tomogram. This is large enough that a rotation or change in twist of the actin filament should be visible in our reconstructions if it occurred, but it is not.

Generally, motility assays are performed with the motor bound to a substrate via the rod domain (S2 plus LMM), which would argue that the S1–S2 junction is comparatively immobile and the actin filament free to move during these assays. If the actin filament were fixed as it appears to be in IFM and the S1–S2 junction free to move on its S2 tether, then the same conformational change in the myosin head that causes left hand twirling of free F-actin would rotate the lever arm anticlockwise or right handed with respect to the thin filament to produce myosin head straightening as observed.

Beausang et al. [84] indicated that the visibility of actin filament rotation in motility assays depends on the number of rigor bound heads providing a drag force to slow filament movement. In isometrically contracting muscle, not only are there many strongly bound heads, but there is an external load to prevent the fibers from shortening. The effect of a torque on the actin filament as a component of the working stroke would by inference be most visible in isometric contraction.

#### Effects on the Thick and Thin Filaments

Forces or components of forces that alter the lever arm azimuth must also impose torsional forces on the thick and thin filaments. Neither the thin filaments, anchored at the Z-disk nor the thick filaments, which are bipolar, can rotate as rigid bodies; they can only change their helical twist in response to an applied torque. For the thin filaments this is not as absolute as for thick filaments since it depends on the compliance of actin crosslinks in the Z-disk. The rotation of the MD on actin during the weak-to-strong transition produces an anticlockwise torque (looking Z-wards) on both the thick and thin filaments (Fig. 10A–C). This torque has opposite effects on the thick and thin filaments because their fixed points are at opposite ends of the half sarcomere. An anticlockwise torque on the thick filament during the weak-to-strong transition, would unwind a right handed helix, but the same torque on the actin filament would over wind a right handed helix. Conversely, an active azimuthal component to the working stroke would apply a clockwise torque to the thick filaments which would over wind a right handed helix and if applied to the thin filament unwind a right handed helix.

There is no evidence in the present tomograms or in any published data from IFM of a change in the half pitch of the thin filament which would be a necessary consequence of any torsional rotations in situ. The thin filament azimuth in our tomograms of active contraction appears virtually identical to that found in other states of IFM investigated by electron microscopy alone [86], [87] and by ET [27], [28]. If a change in thin filament pitch occurred in the present data, the repeat alignment scheme, which utilized only 180° azimuthal rotations, would have obliterated the Tn density which is congruent with the half pitch, whereas in fact, the alignment scheme if anything enhanced it. Lack of evidence that the thin filament in IFM rotates as a rigid body or undergoes changes in helical structure does not mean that the thin filament must be unmodified by strong binding myosin attachments. It only means that any changes appear to be local and not global.

There is some evidence from X-ray diffraction of helical changes in the thin filament of vertebrate striated muscle during isometric contraction [88], [89]. The changes are much smaller than suggested by the present data. Another report describes helical changes in the thin filament in low tension rigor [90] and suggests that these changes are the result of strong binding itself causing local distortions that are measured on average over the entire filament. No publications have reported changes in helical twist of the thick filament during isometric contraction although changes in axial spacings in vertebrate striated muscle are widely known [88], [89].

Perhaps the lack of visible effects on the helical structure of the filaments is due to the differing sense of the two hypothesized torques; the weak to strong transition produces a clockwise torque, twirling results from an anticlockwise torque. More likely the thick and thin filaments are too stiff to be significantly altered by these forces and most of the torsional force is dissipated by S2 and the myosin lever arm.

### Conclusion

We have shown multiple myosin head structures in isometrically contracting muscle consistent with both weak binding and force producing cross-bridges. We can infer from these structures that the weak to strong transition involves largely azimuthal movements of the myosin MD and consequent changes in the lever arm. This agrees with X-ray diffraction observations of the weak to strong transition in which changes in intensity occur largely on the 38.7 nm actin layer line which is sensitive to increases in stereospecific binding, on the equatorial reflections which are sensitive to azimuthal and radial changes in structure and with minimal changes in the 14.5 nm meridional reflection, which is sensitive to lever arm or MD axial orientation [22]. Both our weak binding and strong binding bridges have a symmetrical lever arm axial angle distribution with a mean near 90° while the MD orientation differences are largely azimuthal, qualities that would translate to little change in the 14.5 meridional intensity and large changes in the 38.7 nm actin layer line as tension increases. The 1,1 reflection which measures myosin attachment to actin could remain roughly constant but the large azimuthal changes in the lever would affect the 1,0 intensity. Our strongly biased azimuthal lever arm distribution can be explained by an azimuthal movement of the MD combined with some azimuthal component to the working stroke. That the transition from weak to strong binding is largely azimuthal makes it possible for cross-bridges to cycle in place with little change in axial angle when converting from weak to strong binding [36].

## Materials and Methods

### Rapid Freezing and Freeze Substitution

Rapid freezing with simultaneous monitoring of fiber tension and stiffness was performed on a Heuser Cryopress freezing head [33]. Specific modifications made to the freezing head for this work have been described in detail as have specifics of the specimen manipulation prior to and subsequent to freezing [19], [91].

### Electron Tomography

Details of all the data analysis procedures have been described [37] but a brief summary is given here. Two tilt series covering ±72° at ∼90° relative orientation were recorded from half sarcomeres on a FEI CM300-FEG electron microscope using a Gatan Model 670 High Tilt Analytical Holder and a TVIPS F224 2k×2k charge coupled device camera. Each tilt series consisting of ∼100 images was recorded from regions where longitudinal thin sections contained single myosin and actin filament (myac) layers [35]. Tilt angles within each tilt series were calculated according to the Saxton scheme [92]. The two tilt series were first independently aligned using marker-free alignment [93] and then merged by patch correlation and volume warp using IMOD [94] to produce the raw, dual axis tomogram. The pixel size was internally calibrated using the axial 116 nm period. Although we collected and merged two dual-axis raw tomograms, only one contained an appreciable number, 515, of well centered repeats.

### Repeat Subvolume Processing

Repeats spaced 38.7 nm apart axially and containing a 60.7 nm axial length of the actin filaments, their bound cross-bridges and adjacent thick filament segments were centered on the actin target zones. Alignment error between the raw repeats was minimized by choosing as the reference the single large structure, the thin filament, which was in common to all repeats. A global average of all extracted raw repeats was used as the initial reference and was followed by MDA and multireference alignment. After several cycles of multireference alignment, the final alignment used a single reference as it was desirable to fit one atomic model to the thin filament for all class averages and raw repeats that would be used for all subsequent model building.

### Multivariate Data Analysis and Repeat Reassembly

The purpose of MDA is to sort the highly variable cross-bridge forms within the repeats into self-similar groups. A key part of MDA is the generation of Boolean masks, which select a set of contiguous voxels defining a region of the repeat within which patterns of density will be identified. To retain the greatest variation in structure possible, we generated several classification masks. Two of these selected myosin heads bound to either the left or right side of the actin target zones; these are the primary class averages. Four masks were used for the troponin region, four masks were specific for actins outside of the target zone and two were used for the surface of the thick filament to verify the lever arm positions. Class averages from each classification were subsequently reassembled to make composite class averages [37]. The classification clusters repeats according to the features within the specific mask, but averaging was always carried out using the entire repeat.

Because of the complex manner in which the individual repeats are reassembled, conventional methods of resolution determination, such as the Fourier Shell Correlation are meaningless. However, a qualitative measure of the resolution can be obtained by the fact that the helix of actin subunits can be observed, which requires at least 5.5 nm resolution. The resolution of the previous work was limited to 12. 9 nm so the improvement is at least a factor of 2–3.

### Quasiatomic Models

Quasiatomic models were built in an hierarchical fashion with modifications from earlier studies using rigor IFM fibers [25], [26]. The F-actin atomic model was constructed of 16 G-actin subunits built with the 28/13 helical structure appropriate to IFM thin filaments [95]. A 16 subunit filament was chosen so that two pairs of troponin models [96] could be mounted onto both ends of the filament. We also built onto this actin filament a pair of tropomyosin molecules in the high [Ca^2+^] position [43]. Although we do not resolve tropomyosin in the reconstruction, its presence and plausible location is important for interpreting the different structures.

For cross-bridges whose lever arm orientations were angled toward the rigor configuration, we used an atomic model adapted from the Holmes et al. rigor acto/S1 complex [5] (available at ftp://149.217.48.3/pub/holmes). For cross-bridges whose lever arms appeared to be perpendicular to the thin filament or were angled opposite to rigor, we used the transition state of scallop myosin S1 [10]. The MD of the scallop S1 structure was pre-aligned to the Holmes rigor MD position as the starting model. We evaluated the fitting of the MD first. If the starting model's MD fit the density well, it was kept in this effectively strong binding position, and only the lever arm was adjusted using the following pivot points: residue 710 (or 706), 780 (or 775) and 806 (or 801) in the Holmes S1 model (or scallop S1 model). Otherwise, we fit the MD by first moving the entire starting model as a single rigid body, then adjusted its lever arm position if necessary. Manual fitting was done using the X-ray crystallography model fitting program O [97].

Models were built separately into each left-side target-zone average and each right side target-zone average giving 20 atomic models for each. These were then combined as necessary to produce all of the complete quasiatomic models. This was also done for the troponin bridges. The position of the head-rod junction of the cross-bridges was checked in two ways, either against the density of the raw repeat subvolume or against a column average of the thick filament surface classification. Adjustments were made if indicated.

Figures and movies were constructed using CHIMERA [98].

## Supporting Information

Table S1 Expanded summary of weak attachment models fitted in primary mask class averages.
(0.10 MB DOC)

Movie S1 This is a movie of the quasi atomic model shown in Figure 3A. The coloring scheme is the same as for Figure 3. All movies were made in chimera.
(8.33 MB MOV)

Movie S2 This is a movie of the quasi atomic model shown in Figure 3B. The coloring scheme is the same as for Figure 3. All movies were made in Chimera.
(8.22 MB MOV)

Movie S3 Quicktime movie of Figure 3C. The coloring scheme is the same as for Figure 3. All movies were made in chimera.
(8.48 MB MOV)

Movie S4 Quicktime movie of Figure 3D. The coloring scheme is the same as for Figure 3. All movies were made in chimera.
(8.43 MB MOV)

Movie S5 Quicktime movie of Figure 3E. The coloring scheme is the same as for Figure 3. All movies were made in chimera.
(9.16 MB MOV)

Movie S6 Quicktime movie of Figure 3F. The coloring scheme is the same as for Figure 3. All movies were made in chimera.
(8.84 MB MOV)

Movie S7 Axial diffusion of Type 2 weak binding bridges. This movie shows Z-ward diffusion of a Type 2 weak binding myosin head as might occur during sarcomere shortening when the target zone moves M-ward. The myosin heavy chain is colored magenta to indicate a weak binding state. The myosin head starts out attached to TM in the region of actin subunit F. It then diffuses Z-ward until reaching actin subunit J at which point it is now positioned as a Type 1 weak binding myosin head and can begin the weak-to-strong transition, which largely involves azimuthal movements. When strong binding occurs, signified by change of color to red, the lever arm then moves Z-ward to complete the power stroke. Morphing done using Chimera.
(8.24 MB MOV)

Movie S8 This movie illustrates how S2 can affect the conformation of a myosin head during the weak-to-strong transition followed by a working stroke. An 11 nm long segment of coiled-coil has been attached at the S1–S2 junction. The S2 segment is assumed to be compliant but its origin at the myosin filament backbone is considered fixed; the myosin head is assumed to be non-compliant. The myosin head is initially bound weakly (signified by the magenta colored myosin heavy chain). The weak-to-strong transition involves largely azimuthal movements on its actin subunit (subunit K in this case). When strong binding occurs, signified by the change in heavy chain color to red, the S2 is angled which will result in an azimuthal component to the working stroke.
(9.25 MB MOV)

Movie S9 This movie illustrates a second way that S2 can affect the conformation of a myosin head during the weak-to-strong transition followed by a working stroke. Similar to Movie S8 above, an 11 nm long segment of coiled-coil has been attached at the S1-S2 junction. The S2 segment is assumed to be noncompliant with its origin at the myosin filament backbone fixed; the myosin head is assumed to be compliant. The myosin head is initially bound weakly (signified by the magenta colored heavy chain). The weak-to-strong transition involves largely azimuthal movements on its actin subunit (subunit K in this case) but the noncompliance of the S2 causes the lever arm to bend azimuthally. When strong binding occurs, signified by the change in heavy chain color to red, the S2 is already aligned with the filament axis and the working stroke is executed along the axial direction.
(8.63 MB MOV)

Figure S1 This powerpoint file contains the panels of Figure 10 arranged in an animated sequence that enables the reader to view the changes when superimposed on one another.
(0.06 MB PPT)

## References

[1] Geeves MA, Holmes KC (2005). The molecular mechanism of muscle contraction.. Adv Protein Chem.

[2] Geeves MA, Conibear PB (1995). The role of three-state docking of myosin S1 with actin in force generation.. Biophys J.

[3] Cooke R (1986). The mechanism of muscle contraction.. CRC Crit Rev Biochem.

[4] Rayment I, Rypniewsky WR, Schmidt-Bäse K, Smith R, Tomchick DR (1993). Three-dimensional structure of myosin subfragment-1: a molecular motor.. Science.

[5] Holmes KC, Angert I, Kull FJ, Jahn W, Schroder RR (2003). Electron cryo-microscopy shows how strong binding of myosin to actin releases nucleotide.. Nature.

[6] Holmes KC, Popp D, Gebhard W, Kabsch W (1990). Atomic model of the actin filament.. Nature.

[7] Rayment I, Holden HM, Whittaker M, Yohn CB, Lorenz M (1993). Structure of the actin-myosin complex and its implications for muscle contraction.. Science.

[8] Holmes KC, Geeves MA (2000). The structural basis of muscle contraction.. Philos Trans R Soc Lond B Biol Sci.

[9] Dominguez R, Freyzon Y, Trybus KM, Cohen C (1998). Crystal structure of a vertebrate smooth muscle myosin motor domain and its complex with the essential light chain: visualization of the pre-power stroke state.. Cell.

[10] Houdusse A, Szent-Gyorgyi AG, Cohen C (2000). Three conformational states of scallop myosin S1.. Proc Natl Acad Sci U S A.

[11] Coureux PD, Sweeney HL, Houdusse A (2004). Three myosin V structures delineate essential features of chemo-mechanical transduction.. EMBO J.

[12] Coureux PD, Wells AL, Menetrey J, Yengo CM, Morris CA (2003). A structural state of the myosin V motor without bound nucleotide.. Nature.

[13] Smith CA, Rayment I (1996). X-ray structure of the magnesium(II).ADP.vanadate complex of the *Dictyostelium discoideum* myosin motor domain to 1.9 Å resolution.. Biochemistry.

[14] Whittaker M, Wilson-Kubalek EM, Smith JE, Faust L, Milligan RA (1995). A 35-Å movement of smooth muscle myosin on ADP release.. Nature.

[15] Volkmann N, Liu H, Hazelwood L, Krementsova EB, Lowey S (2005). The structural basis of myosin V processive movement as revealed by electron cryomicroscopy.. Mol Cell.

[16] Yanagida T, Esaki S, Iwane AH, Inoue Y, Ishijima A (2000). Single-motor mechanics and models of the myosin motor.. Philos Trans R Soc Lond B Biol Sci.

[17] Yanagida T, Kitamura K, Tanaka H, Hikikoshi Iwane A, Esaki S (2000). Single molecule analysis of the actomyosin motor.. Curr Opin Cell Biol.

[18] Molloy JE, Burns JE, Kendrick-Jones J, Tregear RT, White DCS (1995). Movement and force produced by a single myosin head.. Nature.

[19] Taylor KA, Schmitz H, Reedy MC, Goldman YE, Franzini-Armstrong C (1999). Tomographic 3-D reconstruction of quick frozen, Ca^2+^-activated contracting insect flight muscle.. Cell.

[20] Esaki S, Ishii Y, Nishikawa M, Yanagida T (2007). Cooperative actions between myosin heads bring effective functions.. Biosystems.

[21] Tsaturyan AK, Bershitsky SY, Burns R, Ferenczi MA (1999). Structural changes in the actin-myosin cross-bridges associated with force generation induced by temperature jump in permeabilized frog muscle fibers.. Biophys J.

[22] Bershitsky SY, Tsaturyan AK, Bershitskaya ON, Mashanov GI, Brown P (1997). Muscle force is generated by myosin heads stereospecifically attached to actin.. Nature.

[23] Ostap EM, Barnett VA, Thomas DD (1995). Resolution of three structural states of spin-labeled myosin in contracting muscle.. Biophys J.

[24] Frank J (2006). Three-Dimensional Electron Microscopy of Macromolecular Assemblies - Visualization of Biological Molecules in Their Native State..

[25] Liu J, Reedy MC, Goldman YE, Franzini-Armstrong C, Sasaki H (2004). Electron tomography of fast frozen, stretched rigor fibers reveals elastic distortions in the myosin crossbridges.. J Struct Biol.

[26] Liu J, Wu S, Reedy MC, Winkler H, Lucaveche C (2006). Electron tomography of swollen rigor fibers of insect flight muscle reveals a short and variably angled S2 domain.. J Mol Biol.

[27] Schmitz H, Reedy MC, Reedy MK, Tregear RT, Winkler H (1996). Electron tomography of insect flight muscle in rigor and AMPPNP at 23°C.. J Mol Biol.

[28] Schmitz H, Reedy MC, Reedy MK, Tregear RT, Taylor KA (1997). Tomographic three-dimensional reconstruction of insect flight muscle partially relaxed by AMPPNP and ethylene glycol.. J Cell Biol.

[29] Pringle JW (1978). The Croonian Lecture, 1977. Stretch activation of muscle: function and mechanism.. Proc R Soc Lond B Biol Sci.

[30] Linari M, Reedy MK, Reedy MC, Lombardi V, Piazzesi G (2004). Ca-activation and stretch-activation in insect flight muscle.. Biophys J.

[31] Heinrich B (1996). The Thermal Warriors.. Strategies of Insect Survival.

[32] Hirose K, Franzini-Armstrong C, Goldman YE, Murray JM (1994). Structural changes in muscle crossbridges accompanying force generation.. J Cell Biol.

[33] Hirose K, Lenart TD, Murray JM, Franzini-Armstrong C, Goldman YE (1993). Flash and smash: Rapid freezing of muscle fibers activated by photolysis of caged ATP.. Biophys J.

[34] Lenart TD, Murray JM, Franzini-Armstrong C, Goldman YE (1996). Structure and periodicities of crossbridges in relaxation and during contraction initiated by photolysis of caged calcium.. Biophys J.

[35] Reedy MK (1968). Ultrastructure of Insect Flight Muscle. I. Screw Sense and Structural Grouping in the Rigor Cross-bridge Lattice.. J Mol Biol.

[36] Tregear RT, Reedy MC, Goldman YE, Taylor KA, Winkler H (2004). Cross-bridge number, position, and angle in target zones of cryofixed isometrically active insect flight muscle.. Biophys J.

[37] Wu S, Liu J, Reedy MC, Winkler H, Reedy MK (2009). Methods for identifying and averaging variable molecular conformations in tomograms of actively contracting insect flight muscle.. J Struct Biol.

[38] Koster AJ, Chen H, Sedat JW, Agard DA (1992). Automated microscopy for electron tomography.. Ultramicroscopy.

[39] Cooke R, Crowder MS, Thomas DD (1982). Orientation of spin labels attached to cross-bridges in contracting muscle fibres.. Nature.

[40] Linari M, Dobbie I, Reconditi M, Koubassova N, Irving M (1998). The stiffness of skeletal muscle in isometric contraction and rigor: the fraction of myosin heads bound to actin.. Biophys J.

[41] Linari M, Caremani M, Piperio C, Brandt P, Lombardi V (2007). Stiffness and fraction of Myosin motors responsible for active force in permeabilized muscle fibers from rabbit psoas.. Biophys J.

[42] Baker TS, Johnson JE (1996). Low resolution meets high: towards a resolution continuum from cells to atoms.. Curr Opin Struct Biol.

[43] Poole KJ, Lorenz M, Evans G, Rosenbaum G, Pirani A (2006). A comparison of muscle thin filament models obtained from electron microscopy reconstructions and low-angle X-ray fibre diagrams from non-overlap muscle.. J Struct Biol.

[44] Conibear PB, Geeves MA (1998). Cooperativity between the two heads of rabbit skeletal muscle heavy meromyosin in binding to actin.. Biophys J.

[45] Corrie JE, Brandmeier BD, Ferguson RE, Trentham DR, Kendrick-Jones J (1999). Dynamic measurement of myosin light-chain-domain tilt and twist in muscle contraction.. Nature.

[46] Squire JM, Harford JJ (1988). Actin filament organization and myosin head labelling patterns in vertebrate skeletal muscles in the rigor and weak binding states.. J Muscle Res Cell Motil.

[47] Varriano Marston E, Franzini Armstrong C, Haselgrove JC (1984). The structure and disposition of crossbridges in deep-etched fish muscle.. J Muscle Res Cell Motil.

[48] Molloy JE, Burns JE, Sparrow JC, Tregear RT, Kendrick-Jones J (1995). Single-molecule mechanics of heavy meromyosin and S1 interacting with rabbit or *Drosophila* actins using optical tweezers.. Biophys J.

[49] Rief M, Rock RS, Mehta AD, Mooseker MS, Cheney RE (2000). Myosin-V stepping kinetics: a molecular model for processivity.. Proc Natl Acad Sci U S A.

[50] Rock RS, Rice SE, Wells AL, Purcell TJ, Spudich JA (2001). Myosin VI is a processive motor with a large step size.. Proc Natl Acad Sci U S A.

[51] Steffen W, Smith D, Simmons R, Sleep J (2001). Mapping the actin filament with myosin.. Proc Natl Acad Sci U S A.

[52] Nishikawa S, Homma K, Komori Y, Iwaki M, Wazawa T (2002). Class VI myosin moves processively along actin filaments backward with large steps.. Biochem Biophys Res Commun.

[53] Veigel C, Wang F, Bartoo ML, Sellers JR, Molloy JE (2002). The gated gait of the processive molecular motor, myosin V.. Nat Cell Biol.

[54] Haselgrove JC, Reedy MK (1984). Geometrical constraints affecting crossbridge formation in insect flight muscle.. J Muscle Res Cell Motil.

[55] Reedy MK, Reedy MC (1985). Rigor crossbridge structure in tilted single filament layers and flared-X formations from insect flight muscle.. J Mol Biol.

[56] Winkler H, Reedy MC, Reedy MK, Tregear R, Taylor KA (1996). Three-dimensional structure of nucleotide-bearing crossbridges in situ: oblique section reconstruction of insect flight muscle in AMPPNP at 23 degrees C.. J Mol Biol.

[57] Narita A, Yasunaga T, Ishikawa T, Mayanagi K, Wakabayashi T (2001). Ca(2+)-induced switching of troponin and tropomyosin on actin filaments as revealed by electron cryo-microscopy.. J Mol Biol.

[58] Bullard B, Bell J, Craig R, Leonard K (1985). Arthrin: a new actin-like protein in insect flight muscle.. J Mol Biol.

[59] Galkin VE, Orlova A, Lukoyanova N, VanLoock MS, Haag P (2003). The location of ubiquitin in Lethocerus arthrin.. J Mol Biol.

[60] Burgess S, Walker M, Knight PJ, Sparrow J, Schmitz S (2004). Structural studies of arthrin: monoubiquitinated actin.. J Mol Biol.

[61] Leonard K, Bullard B, Vigoreaux JO (2006). The thin filament in insect flight muscle.. Nature's Versatile Engine: Insect Flight Muscle Inside and Out.

[62] Brunello E, Reconditi M, Elangovan R, Linari M, Sun YB (2007). Skeletal muscle resists stretch by rapid binding of the second motor domain of myosin to actin.. Proc Natl Acad Sci U S A.

[63] Huxley AF, Simmons RM (1971). Proposed mechanism of force generation in striated muscle.. Nature.

[64] Reedy MK, Linari M, Piperio C, Piazzesi G (1998). Tension transients in single fibres from insect flight muscle.. Pflugers Arch.

[65] Lombardi V, Piazzesi G, Linari M (1992). Rapid regeneration of the actin-myosin power stroke in contracting muscle.. Nature.

[66] Farman GP, Miller MS, Reedy MC, Soto-Adames FN, Vigoreaux JO (2009). Phosphorylation and the N-terminal extension of the regulatory light chain help orient and align the myosin heads in Drosophila flight muscle.. J Struct Biol.

[67] AL-Khayat HA, Hudson L, Reedy MK, Irving TC, Squire JM (2003). Myosin head configuration in relaxed insect flight muscle: X-ray modelled resting crossbridges in a pre-powerstroke state are poised for actin binding.. Biophys J.

[68] Juanhuix J, Bordas J, Campmany J, Svensson A, Bassford ML (2001). Axial disposition of myosin heads in isometrically contracting muscles.. Biophys J.

[69] Gu J, Xu S, Yu LC (2002). A model of cross-bridge attachment to actin in the A*M*ATP state based on x-ray diffraction from permeabilized rabbit psoas muscle.. Biophys J.

[70] Frado LL, Craig R (1992). Electron microscopy of the actin-myosin head complex in the presence of ATP.. J Mol Biol.

[71] Katayama E, Ohmori G, Baba N (1998). Three-dimensional image analysis of myosin head in function as captured by quick-freeze deep-etch replica electron microscopy.. Adv Exp Med Biol.

[72] Tyska MJ, Dupuis DE, Guilford WH, Patlak JB, Waller GS (1999). Two heads of myosin are better than one for generating force and motion.. Proc Natl Acad Sci U S A.

[73] Wong WW, Doyle TC, Reisler E (1999). Nonspecific weak actomyosin interactions: relocation of charged residues in subdomain 1 of actin does not alter actomyosin function.. Biochemistry.

[74] Walker M, Zhang XZ, Jiang W, Trinick J, White HD (1999). Observation of transient disorder during myosin subfragment-1 binding to actin by stopped-flow fluorescence and millisecond time resolution electron cryomicroscopy: evidence that the start of the crossbridge power stroke in muscle has variable geometry.. Proc Natl Acad Sci U S A.

[75] Cooke R (1997). Actomyosin interaction in striated muscle.. Physiol Rev.

[76] Dobbie I, Linari M, Piazzesi G, Reconditi M, Koubassova N (1998). Elastic bending and active tilting of myosin heads during muscle contraction.. Nature.

[77] Irving M, Piazzesi G, Lucii L, Sun YB, Harford JJ (2000). Conformation of the myosin motor during force generation in skeletal muscle.. Nat Struct Biol.

[78] Reedy MC, Reedy MK, Tregear RT (1988). Two attached non-rigor crossbridge forms in insect flight muscle.. J Mol Biol.

[79] Littlefield KP, Ward AB, Chappie JS, Reedy MK, Bernstein SI (2008). Similarities and differences between frozen-hydrated, rigor acto-S1 complexes of insect flight and chicken skeletal muscles.. J Mol Biol.

[80] Huxley HE (1968). Structural difference between resting and rigor muscle; evidence from intensity changes in the low angle equatorial X-ray diagram.. J Mol Biol.

[81] Haselgrove JC, Huxley HE (1973). X-ray evidence for radial cross-bridge movement and for the sliding filament model in actively contracting skeletal muscle.. J Mol Biol.

[82] Brenner B, Yu LC (1993). Structural changes in the actomyosin cross-bridges associated with force generation.. Proc Natl Acad Sci U S A.

[83] Ferenczi MA, Bershitsky SY, Koubassova N, Siththanandan V, Helsby WI (2005). The “roll and lock” mechanism of force generation in muscle.. Structure.

[84] Beausang JR, Schroeder HW, Nelson PC, Goldman YE (2008). Twirling of actin by myosins II and V observed via polarized TIRF in a modified gliding assay.. Biophys J.

[85] Vilfan A (2009). Twirling motion of actin filaments in gliding assays with nonprocessive Myosin motors.. Biophys J.

[86] Reedy MK, Goody RS, Hofmann W, Rosenbaum G (1983). Co-ordinated electron microscopy and X-ray studies of glycerinated insect flight muscle. I. X-ray diffraction monitoring during preparation for electron microscopy of muscle fibers fixed in rigor, in ATP and in AMPPNP.. J Muscle Res Cell Motil.

[87] Reedy MC, Reedy MK, Goody RS (1987). The structure of insect flight muscle in the presence of AMPPNP.. J Muscle Res Cell Motil.

[88] Huxley HE, Stewart A, Sosa H, Irving T (1994). X-ray diffraction measurements of the extensibility of actin and myosin filaments in contracting muscle.. Biophys J.

[89] Wakabayashi K, Sugimoto Y, Tanaka H, Ueno Y, Takezawa Y (1994). X-ray diffraction evidence for the extensibility of actin and myosin filaments during muscle contraction.. Biophys J.

[90] Tsaturyan AK, Koubassova N, Ferenczi MA, Narayanan T, Roessle M (2005). Strong binding of myosin heads stretches and twists the actin helix.. Biophys J.

[91] Taylor KA, Wu S, Reedy MC, Reedy MK, McIntosh JR (2007). Imaging actomyosin in situ.. Cellular Electron Microscopy.

[92] Saxton WO, Baumeister W, Hahn M (1984). Three-dimensional reconstruction of imperfect two-dimensional crystals.. Ultramicroscopy.

[93] Winkler H, Taylor KA (2006). Accurate marker-free alignment with simultaneous geometry determination and reconstruction of tilt series in electron tomography.. Ultramicroscopy.

[94] Mastronarde DN (1997). Dual-axis tomography: an approach with alignment methods that preserve resolution.. J Struct Biol.

[95] Holmes KC, Tregear RT, Barrington Leigh J (1980). Interpretation of the low angle X-ray diffraction from insect muscle in rigor.. Proc Roy Soc Lond B Biol Sci.

[96] Pirani A, Vinogradova MV, Curmi PM, King WA, Fletterick RJ (2006). An atomic model of the thin filament in the relaxed and Ca^2+^-activated states.. J Mol Biol.

[97] Jones TA, Zou JY, Cowan SW, Kjeldgaard M (1991). Improved methods for building protein models in electron density maps and the location of errors in these models.. Acta Crystallog sect A.

[98] Pettersen EF, Goddard TD, Huang CC, Couch GS, Greenblatt DM (2004). UCSF Chimera—a visualization system for exploratory research and analysis.. J Comput Chem.

