# Supplementary material for: Electron Tomography of Cryofixed, Isometrically Contracting Insect Flight Muscle Reveals Novel Actin-Myosin Interactions
Source: PLoS One. 2010 Sep 9;5(9):e12643. doi: 10.1371/journal.pone.0012643 (PMC2936580; doi:10.1371/journal.pone.0012643)
Supplement: Table S1 — Expanded summary of weak attachment models fitted in primary mask class averages. (0.10 MB DOC) [file pone.0012643.s001.doc]

Table 1

Summary of weak attachment models fitted in primary mask class averages.

| Repeat # | # of Members | Actin Label | Type | Total Displacement (nm)^†^ | Axial Coordinate of MD center (nm)^§^ | Radius  of MD center (nm)^§^ | Azimuthal Angle of MD center (°)^§^ | Axial Coordinate Cα of C703 (nm) | Radius Cα of C703 (nm) | Azimuth Cα of C703 (°) |
| --- | --- | --- | --- | --- | --- | --- | --- | --- | --- | --- |
| 298 | 22 | F | 2 | 4.94 | 2.13 | 6.87 | -27.9 | 13.2 | 57.5 | -51.2 |
| 343 | 26 | F | 2 | 5.49 | 2.11 | 7.42 | -25.5 | 15.4 | 71.3 | -47.9 |
| 224 | 15 | F | 2 | 5.97 | 2.06 | 7.10 | -19.0 | 14.8 | 60.7 | -42.1 |
| 289 | 33 | G | 2 | 4.55 | 5.13 | 7.49 | -46.3 | 43.2 | 71.7 | -68.2 |
| 311 | 24 | G | 2 | 4.29 | 1.99 | 7.65 | -38.1 | 14.2 | 75.3 | -59.7 |
| 126 | 19 | H | 2 | 4.30 | 3.72 | 6.98 | -37.9 | 31.5 | 64.2 | -62.0 |
| 107 | 24 | H | 2 | 2.76 | 2.02 | 7.39 | -51.2 | 14.4 | 77.0 | -72.9 |
| 117 | 26 | H | 1 | 1.52 | 2.02 | 6.87 | -83.9 | 14.5 | 80.1 | -104.0 |
| 348 | 27 | H | 1 | 0.97 | 1.62 | 6.34 | -64.3 | 8.1 | 69.1 | -88.2 |
| 246 | 46 | H | 1 | 0.73 | 2.02 | 6.72 | -75.8 | 14.5 | 77.1 | -97.2 |
| 356 | 27 | I | 1 | 2.63 | 2.02 | 6.87 | -94.6 | 14.5 | 80.9 | -114.3 |
| 73 | 24 | I | 1 | 0.96 | 2.02 | 6.74 | -78.6 | 14.5 | 76.8 | -100.2 |
| 311 | 22 | I | 1 | 0.70 | 2.02 | 6.79 | -70.5 | 14.5 | 73.8 | -93.3 |
| 126 | 32 | I | 1 | 0.44 | 2.02 | 6.33 | -75.8 | 14.5 | 71.7 | -99.1 |
| 246 | 18 | I | 1 | 0.19 | 2.02 | 6.27 | -73.1 | 14.5 | 67.2 | -98.2 |
| 117 | 26 | J | 1 | 2.49 | 2.02 | 6.69 | -94.0 | 14.5 | 73.3 | -116.8 |
| 118 | 16 | J | 1 | 1.75 | 2.02 | 6.76 | -86.7 | 14.5 | 66.4 | -111.3 |
| 356 | 21 | J | 1 | 1.67 | 2.02 | 6.43 | -87.2 | 14.5 | 66.1 | -112.5 |
| **246* | *46* | *J* | *1* | *1.39* | *2.02* | *6.56* | *-84.1* | *14.5* | *71.8* | *-107.5* |
| 105 | 34 | J | 1 | 0.73 | 2.02 | 6.32 | -78.7 | 14.5 | 65.6 | -104.2 |
| 395 | 21 | J | 1 | 0.57 | 2.02 | 6.18 | -77.4 | 14.5 | 60.1 | -104.5 |
| 336 | 23 | K | 1 | 0.65 | 2.02 | 6.29 | -78.0 | 14.5 | 59.5 | -104.9 |

*Presumed post rigor class average

^†^Relative to a strongly bound MD placed on actin subunit I

^§^The axial coordinate, radius and azimuthal angle for the center of mass (closest Cα is A462) of a strongly bound MD on actin subunit I is 2.02 nm, 6.11 nm, -72.2°. The corresponding data for C703 is 1.45 nm, 7.83 nm and -90.8°.

Note that the reference for the azimuths given in this table is with respect to the axis of the thin filament and is not the same reference used elsewhere, which is given in Figure 5B.

|  |  |  |  |  |
| --- | --- | --- | --- | --- |
|  |  |  |  |  |
|  |  |  |  |  |
|  |  |  |  |  |
|  |  |  |  |  |
|  |  |  |  |  |
|  |  |  |  |  |
