# Supplementary material for: Electron Tomography of Cryofixed, Isometrically Contracting Insect Flight Muscle Reveals Novel Actin-Myosin Interactions
Source: PLoS One. 2010 Sep 9;5(9):e12643. doi: 10.1371/journal.pone.0012643 (PMC2936580; doi:10.1371/journal.pone.0012643)
Supplement: Figure S1 — This powerpoint file contains the panels of Figure 10 arranged in an animated sequence that enables the reader to view the changes when superimposed on one another. (0.06 MB PPT) [file pone.0012643.s011.ppt]

## Slide 1
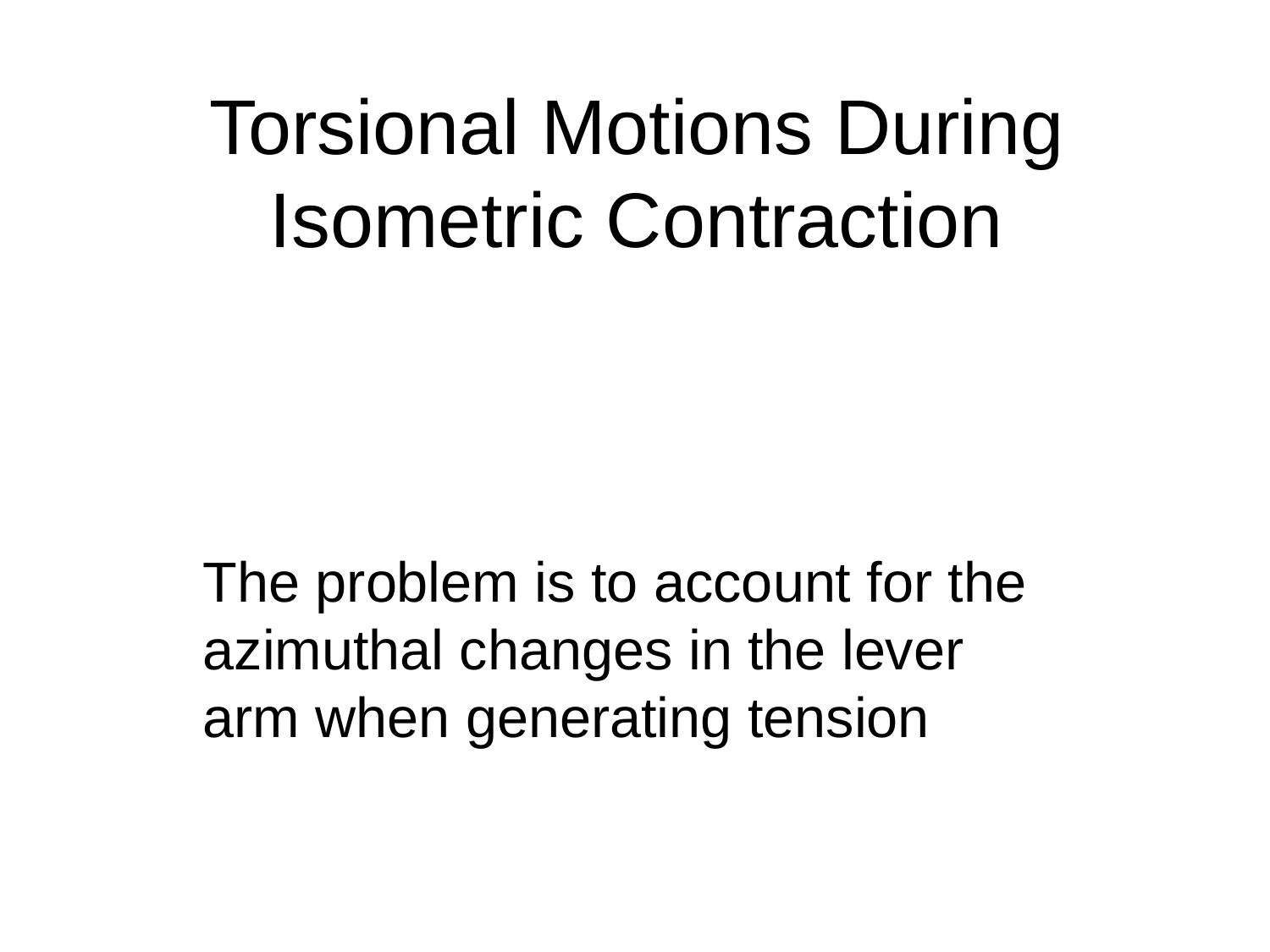

# Torsional Motions During Isometric Contraction
The problem is to account for the azimuthal changes in the lever arm when generating tension

## Slide 2
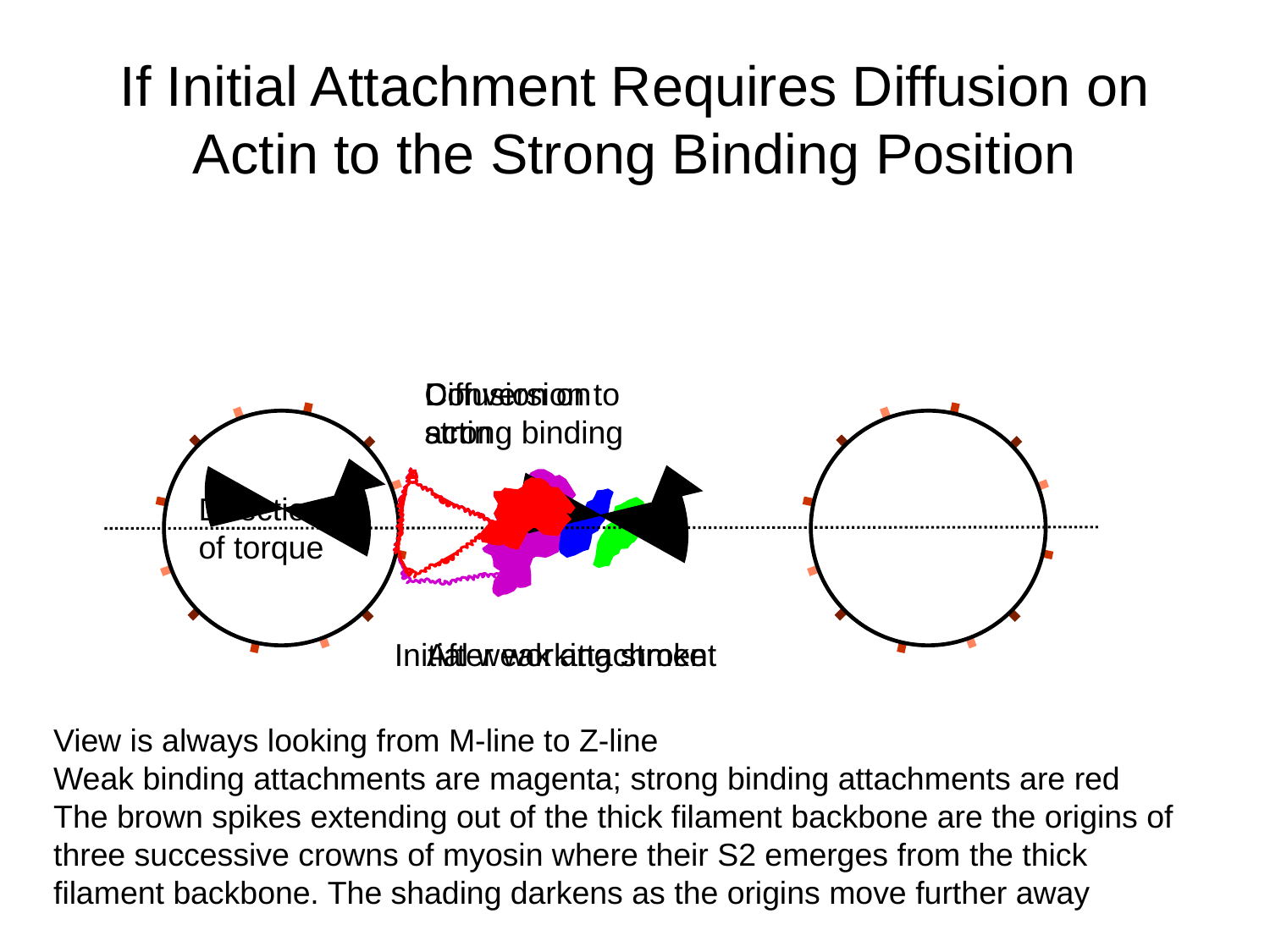

# If Initial Attachment Requires Diffusion on Actin to the Strong Binding Position
Diffusion on
actin
Conversion to
strong binding
Direction
of torque
Initial weak attachment
After working stroke
View is always looking from M-line to Z-line
Weak binding attachments are magenta; strong binding attachments are red
The brown spikes extending out of the thick filament backbone are the origins of three successive crowns of myosin where their S2 emerges from the thick filament backbone. The shading darkens as the origins move further away

## Slide 3
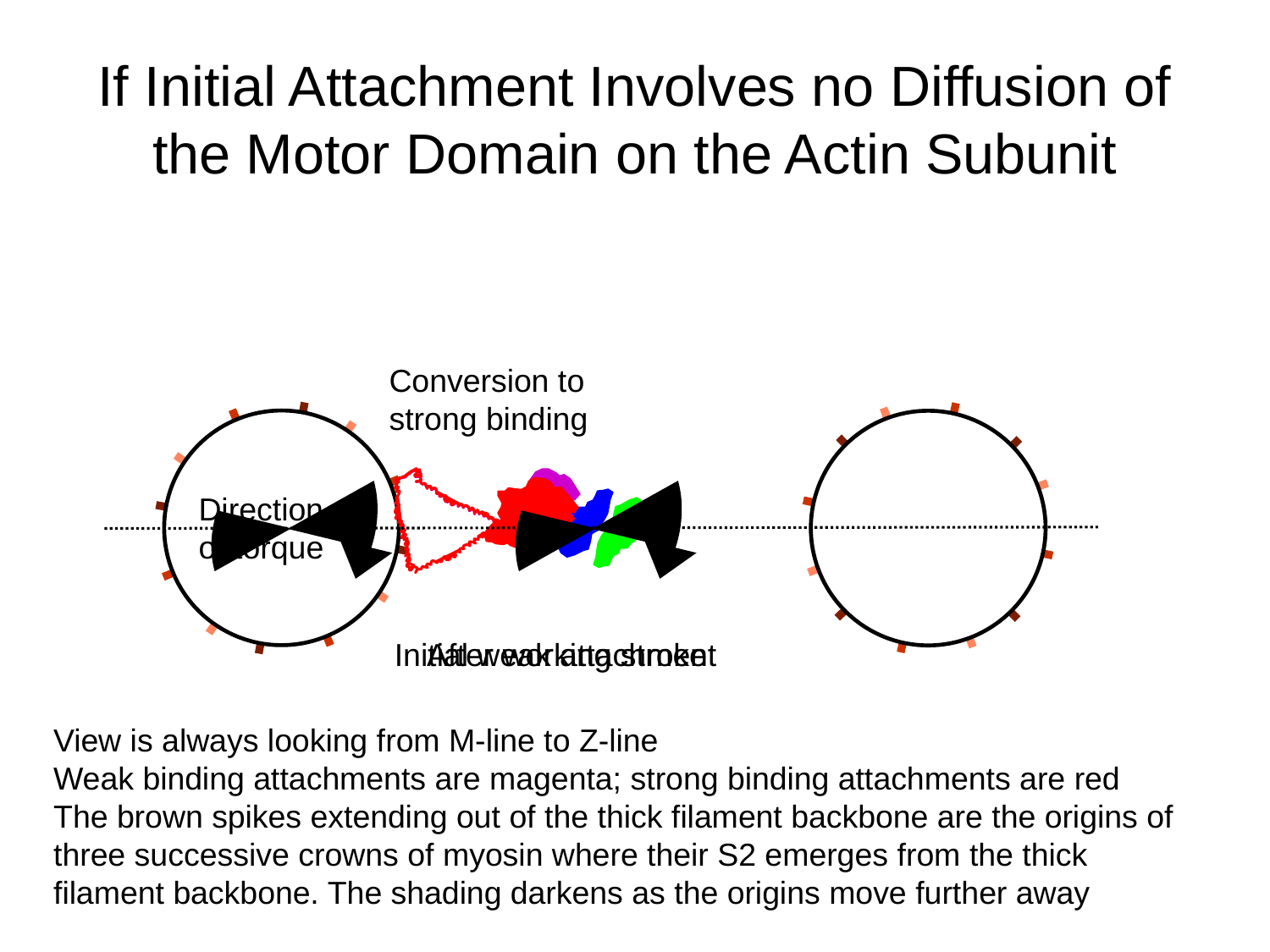

# If Initial Attachment Involves no Diffusion of the Motor Domain on the Actin Subunit
Conversion to
strong binding
Direction
of torque
Initial weak attachment
After working stroke
View is always looking from M-line to Z-line
Weak binding attachments are magenta; strong binding attachments are red
The brown spikes extending out of the thick filament backbone are the origins of three successive crowns of myosin where their S2 emerges from the thick filament backbone. The shading darkens as the origins move further away
